# Performance of verbal autopsy methods in estimating HIV-associated mortality among adults in South Africa

**DOI:** 10.1136/bmjgh-2018-000833

**Published:** 2018-07-03

**Authors:** Aaron S Karat, Noriah Maraba, Mpho Tlali, Salome Charalambous, Violet N Chihota, Gavin J Churchyard, Katherine L Fielding, Yasmeen Hanifa, Suzanne Johnson, Kerrigan M McCarthy, Kathleen Kahn, Daniel Chandramohan, Alison D Grant

**Affiliations:** 1 TB Centre, London School of Hygiene & Tropical Medicine, London, UK; 2 The Aurum Institute, Johannesburg, South Africa; 3 School of Public Health, Faculty of Health Sciences, University of the Witwatersrand, Johannesburg, South Africa; 4 Foundation for Professional Development, Pretoria, South Africa; 5 Division of Public Health, Surveillance and Response, National Institute for Communicable Diseases of the National Health Laboratory Service, Johannesburg, South Africa; 6 MRC/Wits Rural Public Health and Health Transitions Research Unit (Agincourt), School of Public Health, Faculty of Health Sciences, University of the Witwatersrand, Johannesburg, South Africa; 7 INDEPTH Network, Accra, Ghana; 8 Epidemiology and Global Health Unit, Department of Public Health and Clinical Medicine, Umeâ University, Umeâ, Sweden; 9 Department of Disease Control, London School of Hygiene & Tropical Medicine, London, UK; 10 Africa Health Research Institute, Somkhele, South Africa; 11 School of Nursing and Public Health, University of KwaZulu-Natal, Durban, South Africa

**Keywords:** HIV, epidemiology, health systems, medical demography, AIDS

## Abstract

**Introduction:**

Verbal autopsy (VA) can be integrated into civil registration and vital statistics systems, but its accuracy in determining HIV-associated causes of death (CoD) is uncertain. We assessed the sensitivity and specificity of VA questions in determining HIV status and antiretroviral therapy (ART) initiation and compared HIV-associated mortality fractions assigned by different VA interpretation methods.

**Methods:**

Using the WHO 2012 instrument with added ART questions, VA was conducted for deaths among adults with known HIV status (356 HIV positive and 103 HIV negative) in South Africa. CoD were assigned using physician-certified VA (PCVA) and computer-coded VA (CCVA) methods and compared with documented HIV status.

**Results:**

The sensitivity of VA questions in detecting HIV status and ART initiation was 84.3% (95% CI 80 to 88) and 91.0% (95% CI 86 to 95); 283/356 (79.5%) HIV-positive individuals were assigned HIV-associated CoD by PCVA, 166 (46.6%) by InterVA-4.03, 201 (56.5%) by InterVA-5, and 80 (22.5%) and 289 (81.2%) by SmartVA-Analyze V.1.1.1 and V.1.2.1. Agreement between PCVA and older CCVA methods was poor (chance-corrected concordance [CCC] <0; cause-specific mortality fraction [CSMF] accuracy ≤56%) but better between PCVA and updated methods (CCC 0.21–0.75; CSMF accuracy 65%–98%). All methods were specific (specificity 87% to 96%) in assigning HIV-associated CoD.

**Conclusion:**

All CCVA interpretation methods underestimated the HIV-associated mortality fraction compared with PCVA; InterVA-5 and SmartVA-Analyze V.1.2.1 performed better than earlier versions. Changes to VA methods and classification systems are needed to track progress towards targets for reducing HIV-associated mortality,

**Key questions**

**What is already known?**

- Verbal autopsy (VA) is proposed to be used more widely where civil registration and vital statistics systems are weak, but VA methods have not been validated against a quality reference standard for HIV-associated deaths.

**What are the new findings?**

- We used the leading VA interpretation methods to assign causes of death to adults with known HIV status in South Africa.
- Among HIV-positive adults, automated VA estimates of the HIV-associated mortality fraction were much lower than the probable true fraction, though a more recent version of SmartVA-Analyze (V.1.2.1) assigned a higher fraction.
- Automated VA interpretation is likely to result in underestimation of HIV-associated mortality.

**What do the new findings imply?**

- The VA instrument could be enhanced by the addition of an HIV/tuberculosis module; modifications to classifications systems are needed to capture all deaths among HIV-positive people and to differentiate deaths due to HIV-related immunosuppression from other causes.

## Introduction

The Joint United Nations Programme on HIV/AIDS (UNAIDS) aims to reduce the number of AIDS-related deaths from 1.2 million to under 0.5 million by 2020.^1^ Most HIV-related deaths occur in low-income and middle-income countries (LMIC).^2^ Mortality data form the bedrock of health research and policy decision-making, but there are few direct estimates of overall or cause-specific mortality among people living with HIV in LMIC. Global estimates are generated using complex mathematical models and data from several sources, including civil registration and vital statistics (CRVS) systems, seroprevalence surveys, and antenatal and antiretroviral therapy (ART) programmes.^3^ The scarcity of reliable mortality data will make it difficult to track progress towards the ambitious UNAIDS targets.

The direct estimation of HIV-associated mortality is challenging. Death certificates have poor accuracy, particularly in resource-limited settings,^4^ and HIV-related causes may be omitted from death certificates because of stigma.^5–7^ In countries without robust CRVS systems, estimates of cause-specific mortality are often derived, in part, from verbal autopsies (VAs), structured interviews with relatives or carers of deceased individuals, mostly conducted at health and demographic surveillance system (HDSS) sites.^8^ VA interpretation methods assign causes of death (CoD) per International Statistical Classification of Diseases and Related Health Problems (ICD) rules. For demographic purposes, only a single ‘underlying’ CoD is assigned to each decedent; all HIV-associated deaths are thereby included under one of the five ICD categories, defined as, *HIV disease resulting in* (respectively) *infectious and parasitic diseases (B20); malignant neoplasms (B21); other specified diseases (B22); other conditions (B23);* or *unspecified HIV disease (B24)*.^9^

The global roll-out of ART has already had an impact on all-cause mortality in areas of high HIV prevalence^10^ and cause-specific patterns are certain to change as more people receive treatment, live longer, and die of causes other than HIV.^11^ Recent guidelines advocating ART for all people living with HIV will likely accelerate this process,^12^ but current systems do not allow for the enumeration of all deaths among HIV-positive individuals, which is needed to differentiate between deaths due to HIV-related immunosuppression and other causes. While the WHO standardised VA instrument does ask about HIV status, it did not, until recently, ask about ART.^13^ VA has been used extensively in areas of high HIV prevalence, but has not been validated against a robust gold standard for HIV-related and tuberculosis (TB)-related deaths.^14^

Using data from South Africa, we aimed to estimate the sensitivity and specificity of existing WHO VA questions in detecting HIV status and of additional questions in detecting ART initiation, compared with a reference standard of confirmed HIV status and ART initiation from clinical and research data; to estimate the specificity of VA interpretation methods in assigning HIV-associated CoD, compared with confirmed and VA-reported HIV status; and to compare the HIV-associated mortality fractions assigned to HIV-positive adults by different VA interpretation methods.

## Methods

### Parent studies

This study was nested within three large studies conducted in South Africa between 2012 and 2015. (1) ‘TB Fast Track’ was an open, cluster-randomised trial of empirical TB treatment in ambulatory adults with advanced HIV who were not on ART or TB treatment at the point of enrolment, conducted in 24 primary healthcare clinics (PHCs).^15^ (2) ‘XPHACTOR’ was an interventional cohort study investigating the use of Xpert MTB/RIF in a systematic sample of HIV-positive adults attending outpatient clinics for HIV care.^16^ (3) ‘XTEND’ was a cluster-randomised trial evaluating the impact on mortality of Xpert MTB/RIF roll-out; it enrolled HIV-positive and HIV-negative adults who had sputum sent for TB investigation at 40 PHCs.^17^

### HIV-negative individuals

To estimate the specificity of the VA question ‘Was there any diagnosis of HIV/AIDS?’ in detecting HIV status, VAs were conducted among confirmed HIV-negative adults who died in any of the five general hospitals to which TB Fast Track participants were referred. Based on estimated mortality in the three parent studies, 329 VAs were predicted among HIV-positive individuals; VA specificity in assigning HIV-associated deaths was predicted at 90% (based on a large study in Africa).^18^ A target was set of 121 VAs in HIV-negative adults to achieve 95% confidence intervals (CI) of width ±5% around a point estimate of specificity of 90%.

Registers of inpatient deaths were reviewed at hospital mortuaries; all adults who died between June 2014 and October 2015 with a hospital-assigned CoD that was not explicitly HIV-associated, traumatic or maternal were included for further review. Inclusion was initially restricted to individuals aged 18–55 years to be similar in age to HIV-positive decedents, but, due to the small numbers of deaths among young people from causes other than those listed above, this was increased to 18–70 years. The name, sex, dates of birth and death and hospital identifiers of each included adult were recorded; hospital files and the National Health Laboratory Service (NHLS) database were searched for HIV test results. The HIV test date and contact details of next-of-kin, where available, were recorded for those with a negative HIV test (rapid test or ELISA) in the 1 year prior to death and no evidence of a subsequent positive test. Relatives were contacted and a VA conducted per standard procedures outlined below.

### Data collection

VAs were conducted by trained lay-interviewers at the families’ homes or at other locations of their choosing, 1–12 months after death. The WHO 2012 VA instrument was used,^19^ with questions added around ART and treatment for TB (online Supplementary table 1); these questions were asked only to respondents who answered ‘Yes’ to ‘Was there any diagnosis of HIV/AIDS?’ or ‘Was there any diagnosis of tuberculosis?’, respectively. VA interviewers were blinded to causes of death assigned by clinicians at health facilities (unless this was disclosed by the respondent during the VA interview).

10.1136/bmjgh-2018-000833.supp1 Supplementary data

Clinical data to confirm HIV status, ART initiation, TB diagnosis and TB treatment were obtained from clinic and hospital files, parent study databases and the NHLS online database. Data were available for all decedents regarding HIV status and for all HIV-positive decedents regarding ART initiation.

### Data management and cause of death assignment

Quantitative VA data were entered directly into an online database (Mobenzi Technologies, Durban, South Africa) through a cell phone interface; narrative data were captured on paper. Data collected from hospital files and the NHLS database were entered into an EpiData V.3.1 database (The EpiData Association, Odense, Denmark) and data from the parent studies into SQL databases (Bytes Technology Group, Johannesburg, South Africa). VA data were interpreted by both physician-certified VA (PCVA) and computer-coded VA (CCVA);

The PCVA method was based on WHO recommendations, modified in line with changes made at the Medical Research Council/Wits University Agincourt HDSS site, South Africa (online Supplementary figure 1). Two physicians, blinded to all other clinical information, including information on ART obtained through VA, independently reviewed standard VA data and separately assigned CoD using ICD-10 codes. Assigned CoD were compared and, where there were discrepancies, the cases were discussed by the two physicians, aiming for consensus. If a consensus could not be reached, a third, independent physician reviewed the data; if the CoD assigned by physician 3 matched that assigned by physicians 1 or 2, it was considered the final CoD. If no consensus was reached after review by three physicians, the individual was assigned an ‘indeterminate’ CoD. Mortality Medical Data System (MMDS) 2011 software (ftp://ftp.cdc.gov/pub/Health_Statistics/NCHS/Software/mmds/2011) was used to generate a single underlying CoD from multiple CoD assigned by PCVA.

For CCVA interpretation, VA data were mapped to InterVA input variables and fed into InterVA-4.03 and InterVA-5 (http://www.interva.net/), with the prevalence of malaria set to ‘low’ and HIV/AIDS to ‘high’. InterVA cause-specific mortality fractions (CSMFs) were generated by dividing the sum of the likelihoods of each cause category by the sum of likelihoods for all causes after the calculation of the residual indeterminate component, per the InterVA user guide^20^; estimations of specificity and individual agreement used the ‘mostly likely’ assigned cause for comparison. VA data were also mapped to the Population Health Metrics and Research Consortium (PHMRC) full instrument and fed into SmartVA-Analyze V.1.1.1 and V.1.2.1 (http://www.healthdata.org/verbal-autopsy/tools), with ‘Malaria region’ and ‘Free text’ deselected (all versions) and ‘HIV region’ selected (V.1.2.1 only). Narrative data were not provided to SmartVA-Analyze as they were captured on paper.

### Reference definitions

An individual was considered HIV positive if a positive result had been recorded and HIV negative if a negative result had been recorded in the 1 year prior to death without a subsequent positive result. An individual was considered to have initiated ART if this had been recorded in a clinic, hospital or research file; estimation of treatment adherence was not possible based on the data available. Self-report was not considered sufficient evidence of HIV status (positive or negative) or ART initiation. A death due to ICD-10 codes B20–B24 (PCVA; underlying cause), ‘HIV/AIDS’ (InterVA) or ‘AIDS’ (SmartVA-Analyze) was considered HIV-associated; a death from any other cause was considered non-HIV-associated.

### Statistical analysis

**Figure 1:**
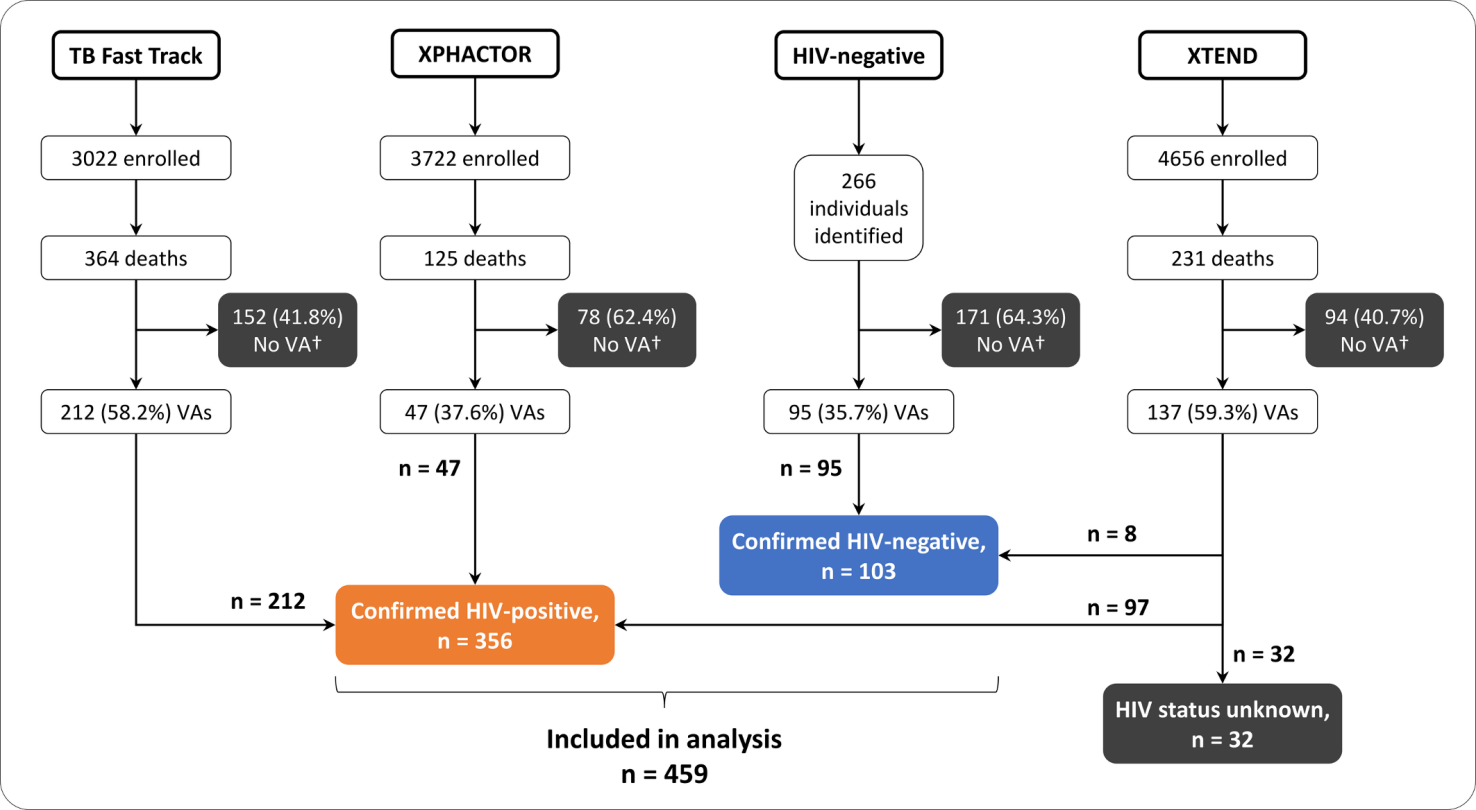
Flow diagram showing numbers enrolled into each of the parent studies*, subsequent deaths, number of VAs completed and numbers of confirmed HIV-positive and HIV-negative individuals included in final analysis. *TB Fast Track and XPHACTOR enrolled only HIV-positive adults; XTEND enrolled HIV-positive and HIV-negative adults being investigated for TB. †VA was attempted but could not be completed. TB, tuberculosis; VA, verbal autopsy.

Individuals with unknown HIV status were excluded from all analyses (figure 1). For estimation of sensitivity and specificity, answers to VA questions of ‘Do not know’ were recoded as ‘No’, to match the methods used by InterVA and SmartVA-Analyze.^21 22^ The sensitivity and specificity of VA questions were calculated with exact binomial 95% CI. The specificities of VA methods in assigning HIV-associated CoD were calculated compared with confirmed HIV status, as defined above, and VA-reported HIV status, to simulate situations in which clinical HIV data are not available.

**Figure 2:**
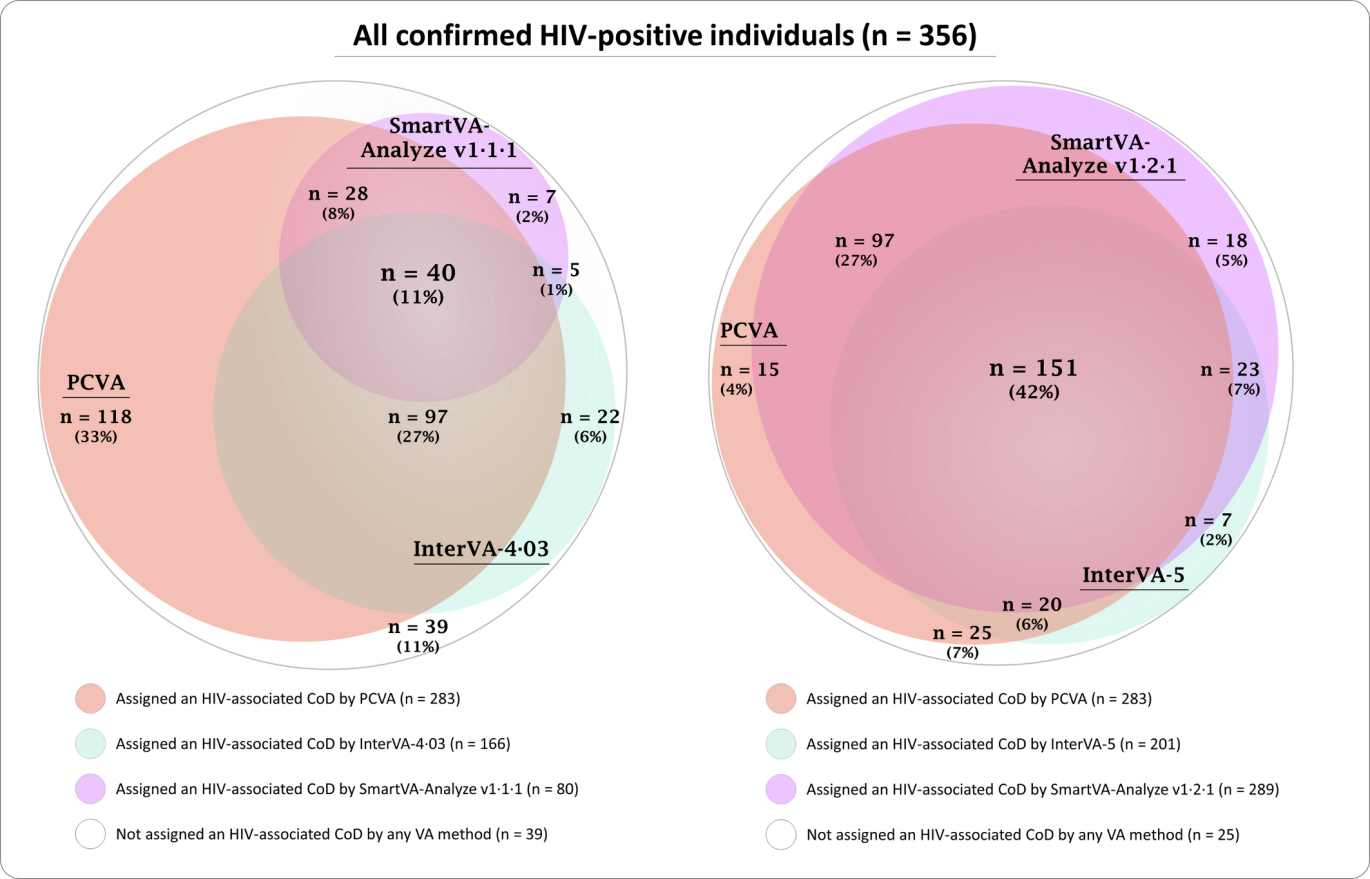
Euler diagram illustrating the number of confirmed HIV-positive individuals assigned HIV-associated CoD by the five VA methods and overlap between methods, using SmartVA-Analyze V.1.1.1 and InterVA-4 (left) and SmartVA-Analyze V.1.2.1 and InterVA-5 (right; n, [%/356]). CoD, cause of death; PCVA, physician-certified verbal autopsy; VA: verbal autopsy.

Reference CoD were not available for all decedents and, because people with HIV may die from many causes, including those unrelated to HIV, sensitivity of VA methods in assigning CoD could not be estimated. Cohen’s kappa (Κ) and chance-corrected concordance (CCC) were used to measure individual-level agreement between VA methods; Lin’s concordance correlation coefficient (ρ_C_), CSMF accuracy, and chance-corrected CSMF accuracy were used to measure population-level agreement based on two possible CoD: HIV-associated and non-HIV-associated, as defined above. In the absence of a clinical reference standard, CCVA CoD were compared with PCVA CoD. All analyses were conducted using Stata V.14. Figure 2 was developed using eulerAPE^23^ and Inkscape (https://inkscape.org) software.

### Ethical considerations

This substudy was approved by the human research ethics committees of the London School of Hygiene & Tropical Medicine and the University of the Witwatersrand. All decedents were assigned numeric identities to ensure anonymity during collection of clinical data and all VA respondents gave written informed consent for interview.

### Role of the funding source

The funders had no role in the design of the study; in the collection, analysis, and interpretation of data; in the writing of the report; or in the decision to submit the paper for publication.

## Results

### Demographics

A total of 986 VAs were attempted; 491 (49.8%) were successfully completed (figure 1). Individuals with unknown HIV status (n=32) were excluded from the analysis. VA data from 459 individuals were included, 356 (77.6%) HIV positive and 103 (22.4%) HIV negative (table 1); 240 (52.3%) decedents were female and the median age was 41.5 (IQR 34–52) years. Demographics are described in table 1; comparison between HIV-positive and HIV-negative individuals showed important differences only in median age (39.6 vs 52.1 years; p<0.001).

**Table 1:** Demographics for all decedents, stratified by confirmed HIV status (n=459)

Characteristic	All with confirmed HIV status (n=459), n (%) or median (IQR)	Confirmed HIV positive (n=356), n (%) or median (IQR)	Confirmed HIV negative (n=103), n (%) or median (IQR)	P values*
Female	240 (52.3)	195 (54.8)	45 (43.7)	0.05
Age at death, years	41.5 (33.6 to 51.5)	39.6 (33.0 to 47.4)	52.2 (42.4 to 60.9)	<0.001
Black African	457 (99.6)	354 (99.4)	103 (100)	0.45
South African national	433 (94.3)	336 (94.4)	97 (94.2)	0.94
Enrolled or hospitalised in periurban area†	330 (71.9)	253 (71.1)	77 (74.8)	0.46
Initiated ART after enrolment‡	117 (25.5)	117 (32.9)	NA	–
Time from				
Enrolment‡ to death, days	80.5 (35 to 161) (n=356)	80.5 (35 to 161)	NA	–
HIV-negative test to death, days	14 (5 to 59) (n=103)	NA	14 (5–59)	–
Death to VA, days	218.5 (106 to 325)	235 (102 to 338)	174 (110 to 271)	0.04

*Kruskal-Wallis or Χ^2^ test, as appropriate.

†Site of enrolment for individuals enrolled to one of the three parent studies; site of hospitalisation for HIV-negative individuals recruited from hospitals.

‡Enrolment into parent study.

ART, antiretroviral therapy; VA, verbal autopsy.

### Sensitivity and specificity of VA questions

The VA question, ‘Was there any diagnosis of HIV/AIDS?’ correctly identified 300/356 HIV-positive individuals and incorrectly identified 6/103 HIV-negative individuals as HIV positive (sensitivity 84.3% [95% CI 80 to 88]; specificity 94.2% [95% CI 88 to 98]; table 2). The question ‘Did the deceased ever take ART?’, added to the WHO VA instrument, was asked to the 306 respondents who answered ‘Yes’ to the HIV diagnosis question: 193/212 individuals who had initiated ART were correctly reported as having done so, but the question was also answered ‘Yes’ for 44/94 individuals who had not initiated ART (sensitivity 91.0% [95% CI 87 to 95]; specificity 53.2% [95% CI 43 to 64]).

**Table 2:** Sensitivity, specificity, and measures of agreement of VA questions regarding HIV status and ART initiation (n=459)

VA question	Answers			Sensitivity*, % (95% CI)	Specificity*, % (95% CI)	Overall agreement*†, %	Κ* (95% CI)	CCC*
	Yes, n (%)	No, n (%)	DNK, n (%)					
Was there any diagnosis of HIV/AIDS? (n=459)	306 (66.7)	111 (24.2)	42 (9.2)	84.3 (80.1 to 87.9)	94.2 (87.8 to 97.8)	86.5	0.68 (0.60 to 0.74)	0.69
Did she/he ever take ART? (n=306)‡	237 (77.5)	50 (16.3)	19 (6.2)	91.0 (86.4 to 94.5)	53.2 (42.6 to 63.6)	81.4	0.48 (0.37 to 0.59)	0.82

* No’ and ‘Do not know’ answers combined for analysis.

†Overall agreement is the proportion considered ‘positive’ or ‘negative’ by both clinical and VA methods (ie, (true positives+true negatives)/total).

‡VA respondents were only asked about ART initiation if they answered ‘Yes’ to HIV question; decedents who were confirmed HIV positive, but were not reported as such by the respondent, were not included in this analysis. Of the 306 individuals reported as HIV positive at VA, 212 (69.3%) had initiated ART and 94 (30.7%) had not.

ART, antiretroviral therapy; CCC, chance-corrected concordance; DNK, do not know; TB, tuberculosis; VA, verbal autopsy; Κ, Cohen’s kappa.

### ART initiation dates

Dates of ART initiation were available from both VA interviews and clinical records for 125 individuals. The median difference between dates obtained from the two sources was 29 (IQR 11–111) days; 90/125 (72.0%) and 108/125 (86.4%) ART initiation dates from VA interviews were within 90 days and 1 year, respectively, of the dates in clinical records. Of the 237 individuals who were reported, at VA, to have been taking ART, 36 (15.1%) were reported not to have been taking it at death and 62 (26.2%) not to have been taking it every day.

### Performance of VA interpretation methods

#### Estimating HIV-associated mortality among HIV-positive adults

Of 356 HIV-positive adults, 283 (79.5%) were assigned an HIV-associated CoD by PCVA; 166 (46.6% as ‘most likely’ cause; CSMF 44.7% when all assigned CoD and associated likelihoods included) by InterVA-4.03; 201 (56.5%; CSMF 54.5%) by InterVA-5; 80 (22.5%) by SmartVA-Analyze V.1.1.1; and 289 (81.2%) by SmartVA-Analyze V.1.2.1 (table 3 and figure 2). These proportions were higher among the 300 individuals reported HIV positive at VA (255 [85%] PCVA; 150 [50%; CSMF 48%] and 178 [59%; CSMF 57%] InterVA-4.03 and InterVA-5; and 69 [23%] and 285 [95%] SmartVA-Analyze V.1.1.1 and V.1.2.1). Individual-level and population-level agreement was poor between PCVA/InterVA-4.03 and PCVA/SmartVA-Analyze V.1.1.1 (CCC<0; CSMF accuracy ≤56%); agreement was better between PCVA and newer versions of InterVA and SmartVA-Analyze (CCC 0.21 and 0.75; CSMF accuracy 65% and 98%; table 3).

**Table 3:** Number of confirmed HIV-positive individuals (n=356) assigned an HIV-associated CoD by five VA methods, stratified by VA-reported HIV status and agreement between PCVA and CCVA methods

VA interpretation method	Number of confirmed HIV-positive individuals assigned an HIV-associated CoD				Agreement with PCVA*				
	All, n (CSMF; %/356)	By VA-reported HIV status			Individual level		Population level		
		Positive, n (CSMF; %/300)	Negative, n (CSMF; %/23)	Do not know†, n (CSMF; %/33)	Κ (95% CI)	CCC	ρ_C_	CSMF accuracy (%)	CCCSMF accuracy (%)
PCVA	283 (79.5)	255 (85.0)	6 (26.1)	22 (66.7)	–	–	–	–	–
InterVA-4.03‡	(44.7)	(47.6)§	(28.0)§	(29.7)§	0.05 (0 to 0.13)¶	−0.03¶	−0.348	56.2	−18.9
InterVA-5‡	(54.5)	(57.4)§	(37.8)§	(39.6)§	0.14 (0.05 to 0.23)¶	0.21¶	0.298	64.6	14.5
SmartVA-Analyze V.1.1.1	80 (22.5)	69 (23.0)	3 (13.0)	8 (24.2)	0.04 (0 to 0.09)	−0.52	−0.998	28.3	−94.8
SmartVA-Analyze V.1.2.1	289 (81.2)	285 (95.0)	1 (4.4)	3 (9.1)	0.33 (0.21 to 0.44)	0.75	0.998	97.9	94.2

*Measures of agreement calculated using two possible causes of death: HIV-associated and non-HIV-associated.

†Answers of’ Do not know’ listed as ‘Negative’ when estimating agreement.

‡InterVA-4 and InterVA-5 CSMFs calculated from all assigned CoD with associated likelihoods.

§CSMF calculated separately per stratum.

¶Measures of individual agreement for InterVA calculated using individuals assigned HIV/AIDS as ‘most likely’ CoD (n=166 for InterVA-4; n=201 for InterVA-5).

CCC, chance-corrected concordance; CCCSMF, chance-corrected cause-specific mortality fraction; CCVA, computer-coded verbal autopsy; CoD, cause of death; CSMF, cause-specific mortality fraction; PCVA, physician-certified verbal autopsy; VA, verbal autopsy; Κ, Cohen’s kappa; ρ_C_, Lin’s concordance correlation coefficient.

#### Specificity in assigning HIV-associated CoD

Among all 459 individuals, PCVA assigned 287 (63%) HIV-associated CoD, compared with 177 (39%; CSMF 37%) by InterVA-4.03, 214 (47%; CSMF 45%) by InterVA-5, and 85 (19%) and 294 (64%) by SmartVA-Analyze V.1.1.1 and V.1.2.1, respectively (table 4). Compared with confirmed HIV status, the specificity of PCVA was 96.1% (95% CI 90 to 99); specificities of CCVA methods were 89.3% (95% CI 82 to 95), 87.4 (95% CI 79 to 93), 95.1% (95% CI 89 to 98), and 95.1% (95% CI 89 to 98) for InterVA-4.03, InterVA-5, and SmartVA-Analyze V.1.1.1 and V.1.2.1, respectively. Specificities of all methods, except SmartVA-Analyze V.1.2.1, which increased to 97%, were lower when compared with VA-reported HIV status, at 81%, 78%, 83%, and 90% for PCVA, InterVA-4.03, InterVA-5, and SmartVA-AnalyzeV.1.1.1, respectively (table 4).

**Table 4:** Number assigned (n=459) and specificity of VA methods in assigning HIV-associated causes of death, compared with confirmed (n=103) and VA-reported (n=153) HIV status

VA method	Number assigned an HIV-associated CoD, n (CSMF; %/459)	Specificity of VA method	
		Based on confirmed serostatus (95% CI)*	Based on VA-reported HIV status (95% CI)†
PCVA	287 (62.5)	96.1 (90.4 to 98.9)	81.0 (73.9 to 86.9)
InterVA-4.03‡	(37.1)	89.3 (81.7 to 94.5)§	83.0 (76.1 to 88.6)§
InterVA-5‡	(44.9)	87.4 (79.4 to 93.1)§	78.4 (71.1 to 84.7)§
SmartVA-Analyze V.1.1.1	85 (18.5)	95.1 (89.0 to 98.4)	90.2 (84.3 to 94.4)
SmartVA-Analyze V.1.2.1	294 (64.1)	95.1 (89.0 to 98.4)	97.4 (93.4 to 99.3)

*n=103 individuals confirmed HIV negative.

†n=153 individuals reported HIV negative or with HIV status unknown.

‡InterVA-4 and InterVA-5 CSMFs calculated from all assigned CoD with associated likelihoods.

§Individuals considered ‘test positive’ if HIV/AIDS assigned as most likely CoD (n=177 for InterVA-4; n=214 for InterVA-5).

CoD, cause of death; CSMF, cause-specific mortality fraction; PCVA, physician-certified verbal autopsy; VA, verbal autopsy.

## Discussion

The VA question about HIV diagnosis showed moderate to high sensitivity and specificity in correctly identifying HIV status. Added questions around ART initiation were also sensitive and a high proportion of VA ART initiation dates were within 3 months of the confirmed date from clinical records. VA interpretation methods differed widely in their estimation of the HIV-associated mortality fraction among confirmed HIV-positive individuals; CCVA methods used until 2016 gave estimates that were likely much lower than the true fraction. All VA interpretation methods showed high specificity in assigning HIV-associated CoD.

### Estimating HIV prevalence and ART initiation

The moderate-to-high sensitivity and specificity of the HIV question in detecting HIV status seen here and in a study conducted in Malawi (sensitivity 83%, specificity 98%)^24^ suggests that VA may be useful in generating estimates of HIV prevalence among deceased individuals. However, sensitivity was lower when tested in a larger, more diverse population: a study conducted across five sub-Saharan African countries from 1990 to 2011 focused primarily on estimating the specificity of InterVA-4 in diagnosing HIV-associated CoD^18^; crude estimates of sensitivity and specificity of the VA instrument in detecting HIV status can, however, be derived from the data presented (sensitivity 33.7% [95% CI 32 to 36], specificity 93.6% [95% CI 93 to 95]). Differences in estimates of sensitivity may be attributable to increased availability of testing and reduced stigma over time, particularly as the multicountry study analysed deaths that occurred over a 20-year period^18^; in the Malawian study, the proportion of respondents reporting knowledge of HIV status increased from 48% in 2003/2004 (n=300) to 99% in 2013/2014 (n=303).^24^ The consistently high specificity suggests that VA is unlikely to overestimate HIV prevalence in high prevalence settings; further evaluations of the VA question are needed, including reanalysis of existing raw VA data from HDSS sites, to assess better its suitability for estimating HIV prevalence in different contexts.

The only other study to have evaluated VA for ART use estimated sensitivity and specificity at 92% and 46%, respectively (Malawi, 2009–2014, n=154).^24^ The low specificity seen also in our study suggests a need to further refine questions around ART; HIV-positive individuals are often prescribed several different drugs and confusion among VA respondents is understandable. Inclusion of variants of these questions in future VA validation studies is recommended.

### Estimating HIV-associated mortality

The high specificity of PCVA and InterVA in assigning HIV-associated CoD is consistent with previous evaluations in settings with high HIV prevalence. The specificity of PCVA has been between 89% and 99% in studies conducted in Uganda,^25^ Tanzania,^26^ and Malawi,^27^ and InterVA-4 has been reported as 76%–90% specific in studies in several African countries.^18 27 28^ No similar evaluations of SmartVA-Analyze were found in the literature.

The consistently high specificity of VA interpretation methods suggest that they are unlikely to overestimate HIV-associated mortality. Estimating the sensitivity of a VA method, however, is challenging even when high-quality data are available, given the inherent uncertainty involved in assigning CoD. Only one study, conducted by our group in South Africa, has compared VA to CoD derived from pathological autopsy in HIV-positive individuals and found that VA methods likely underestimated HIV-associated CoD.^29^ In part because of difficulties faced with ICD coding, there are few direct (autopsy or clinical) estimates of the HIV-associated mortality fraction among HIV-positive adults. A recent systematic review, which included only studies of HIV-positive individuals entirely or mostly on ART (n=19 studies), estimated that 18.5% (95% CI 13 to 24) of deaths in HIV-positive individuals on ART in sub-Saharan countries were due to ‘non-AIDS’ causes (ie, 76%–83% due to AIDS).^11^ This review, however, treated deaths due to ART toxicity as ‘non-AIDS’, which is contrary to WHO guidance^30^ and means that overall HIV-associated mortality may have been underestimated. In populations containing fewer individuals on ART, the HIV-associated mortality fraction is likely to be even higher, as seen in several pathological autopsy studies.^31^ In our study, only PCVA and SmartVA-Analyze V.1.2.1 estimated an HIV-associated mortality fraction close to the figure above (80% and 85%). InterVA-4.03, InterVA-5, and SmartVA-Analyze V.1.1.1 estimated smaller HIV-associated mortality fractions (45%, 55%, and 23%); as a high number of individuals in our study had not initiated ART, these estimates are likely lower than the true HIV-associated mortality fraction in this population.

Plans are underway to integrate VA into CRVS systems,^32^ a proposal made feasible, in part, by the increased efficiency of CCVA methods. VA has been previously used mainly at HDSS sites, where, through trend analysis, systematic errors in estimates could be detected and adjusted for. If used to supplement CRVS data, however, VA findings may directly influence policy and health planning decisions and will need to be more stringently validated for key causes of mortality such as HIV.

In our study, SmartVA-Analyze V.1.2.1 assigned a much higher HIV-associated mortality fraction than V.1.1.1, though the reason for the change and the effect of this on assigning other causes is not clear, as code for the software is not available. It may be that the newer version is guided by reported HIV status: V.1.2.1 assigned an HIV-associated cause to 95% of individuals reported HIV-positive in the VA interview, compared with 23% assigned by V.1.1.1 (data not shown). None of the VA methods evaluated here was provided with data on ART use; simply assigning an HIV-associated cause to all HIV-positive individuals is unlikely to be a viable long-term strategy in areas with high ART coverage. Nevertheless, both older CCVA methods likely allocate insufficient weight to the question about HIV diagnosis, which has now been shown to be sensitive and specific in Malawian^24^ and South African contexts. InterVA assigns CoD based on a probabilistic algorithm, which weights questions per the recommendations of a panel of expert physicians, convened in 2006 and 2011.^33^ In our study, physicians reviewing VA data gave the HIV question high importance, assigning an HIV-associated CoD to 84% of the 306 individuals reported HIV-positive and to 52% of the 42 individuals where the respondent answered, ‘Do not know’ (compared with 49% and 24% by InterVA-4.03; 59% and 33% by InterVA-5; 23% and 21% by SmartVA-Analyze V.1.1.1; and 95% and 7% by SmartVA-Analyze V.1.2.1 [n=459; data not shown]). In part, this is likely because all ‘Do not know’ answers are converted to ‘No’ by these CCVA methods. Along with adjustments to weighting, an ability to differentiate between the two answers may be needed in the interpretation of key questions such as this.

### Limitations and strengths

All individuals included in this analysis were recruited from health facilities (clinics or hospitals) and around 76% died in hospitals (data not shown). This is higher than the proportion of facility-based deaths in South Africa (43% in hospital, 23% at home, 23% in unknown locations in 2016)^34^ and likely in many other countries in sub-Saharan Africa (though these data are not available due to the absence of robust CRVS systems in most of these countries).^7^ This may have led to overestimation of the sensitivity and specificity of questions around HIV diagnosis and ART, as families of individuals who received medical care prior to death are more likely to be familiar with medical terminology.^35^ In addition, 72% of individuals were enrolled or died in urban areas; this limits the generalisability of our findings to more rural parts of South Africa or other countries in sub-Saharan Africa, which have larger rural populations (an estimated 62% of people in the WHO AFRO region were living in rural areas in 2013).^36^ However, the high sensitivity and specificity of the VA HIV question in the Malawian study cited above, which was conducted in an HDSS site,^24^ and the only slight-to-moderate reductions, in our study, in the sensitivity and specificity of VA questions and VA methods (specificity only) in rural versus urban settings (online Supplementary tables 2–4), suggest that these questions and methods may perform similarly in other sub-Saharan African settings, though further evaluation is needed.

The relatively low number of HIV-negative individuals reduced the accuracy of specificity estimates and, despite best efforts, the HIV-positive and HIV-negative groups were not age matched. HIV is generally less likely to be considered as a CoD in older individuals^37^ (though recent data from South Africa suggest this may no longer be appropriate)^38^; the higher median age of HIV-negative decedents may have led to overestimation of specificities in detecting HIV status and assigning HIV-associated CoD. Estimates of sensitivity, however, should not have been affected. Physicians assigning CoD were aware that decedents were likely to have been enrolled in studies of HIV/TB and this may have led to their assigning higher numbers of HIV-associated CoD; VA interviewers were aware of a decedent’s HIV status before the VA was conducted, and this may have affected the way questions around HIV and ART were asked or interpreted during the interview. The most recent WHO VA instrument^39^ was not used for data collection and SmartVA-Analyze was not provided with narrative data, which may have led to suboptimal performance of the software, although the omission of these data will likely have had minimal effect, as the software does not consider the phrases ‘HIV’ or ‘AIDS’ as ‘words of interest’.^22^ Clinical reference CoD were not available for all decedents and the sensitivity of VA methods in assigning HIV-associated CoD could not be assessed.

This study’s strengths include having confirmed HIV status for all decedents included in the analysis; the median time from HIV-negative test to death being 2 weeks, with 75% of HIV-negative individuals tested less than 2 months before death; and using the leading VA interpretation methods, including the most recent versions of SmartVA-Analyze and InterVA, to estimate the HIV-associated mortality fraction among HIV-positive individuals, something not previously done in VA studies.

### Next steps

The UNAIDS goal of less than 0.5 million HIV-related deaths by 2020 calls for an absolute reduction in events,^1^ which will require methods to identify HIV-associated deaths more accurately.^14 40^ Classification systems require alterations that will allow for the identification of all deaths in people with HIV and prediction of possible changes in mortality patterns through increased availability of ART; a flexible system, with shorter or partial revision cycles, would allow for more adaptability in the face of an evolving epidemic. In the short term, other methods may provide useful data for monitoring changes in mortality patterns: a recent study from Mozambique found good agreement between CoD assigned by minimally invasive autopsy (MIA) and complete autopsy.^41 42^ These findings, along with previous evaluations of MIA,^43–45^ suggest it may be useful in providing more accurate CoD data as an adjunct to surveillance at sentinel sites in LMIC.

## Conclusions

VA interpretation methods differed in their estimates of HIV-associated mortality and older CCVA methods underestimated the HIV-associated mortality fraction; estimates from PCVA and revised CCVA methods were closer to the probable true fraction. VA questions were sensitive and specific in detecting HIV status and sensitive in detecting ART initiation. The addition of an HIV/TB module to the VA instrument is recommended; future VA validation studies should be used to trial questions prior to inclusion in the standardised VA instrument. Modifications to classification systems are needed to capture all deaths among HIV-positive people and differentiate deaths due to HIV-related immunosuppression from other causes; closely aligned is a need for more accurate direct estimates of HIV-associated mortality to track progress towards goals set by UNAIDS.
