# Supplementary material for: Performance of verbal autopsy methods in estimating HIV-associated mortality among adults in South Africa
Source: BMJ Glob Health. 2018 Jul 3;3(4):e000833. doi: 10.1136/bmjgh-2018-000833 (PMC6035502; doi:10.1136/bmjgh-2018-000833)
Supplement: Supplementary data [file bmjgh-2018-000833supp001.pdf]

SUPPLEMENTARY INFORMATION

Supplementary table 1. Questions regarding antiretroviral therapy and treatment for TB added to the WHO 2012 VA instrument by the study team and the parent questions to which they were attached

**From WHO 2012 VA instrument**	**Added by study team**	
**Parent question**	**Sub-question(s) (if parent question = 'Yes')**	**Sub-sub-question(s) (if sub-question = 'Yes')**
**Was there any diagnosis of Tuberculosis?**	Did s/he ever take treatment for TB from a clinic/hospital?	When did s/he start TB treatment? (If treated more than once, record the most recent episode)
		Which clinic/health facility did s/he attend most recently (name)?
		Location of clinic/health facility (nearest town)
		Were they still taking TB treatment at the time of death?
		If TB treatment was stopped early why was that?
		Was s/he taking medication every day in the way they s/he was supposed to?
**Was there any diagnosis of HIV/AIDS?**	Did the deceased ever take Antiretroviral Therapy (ART)?	When did s/he start ART?
		Which clinic/health facility did s/he attend most recently (name)?
		Location of clinic/health facility (nearest town)
		Were they still taking ART at the time of death?
		If ART was stopped, why was that?
		Was s/he taking the medication every day?

AIDS: acquired immune deficiency syndrome; ART: antiretroviral therapy; HIV: human immunodeficiency virus; TB: tuberculosis; VA: verbal autopsy; WHO: World Health Organization

Supplementary table 2. Sensitivity and specificity of VA questions about HIV status (n = 459) and ART initiation (n = 306), stratified by sex, age group, area of enrolment/hospitalisation, site of death, and time from death to VA

Group	HIV question*			ART question†		
	N	Sensitivity, % (95 CI)	Specificity, % (95 CI)	N	Sensitivity, % (95 CI)	Specificity, % (95 CI)
**Overall**	**459**	**84.3 (80.1–87.9)**	**94.2 (87.8–97.8)**	**306**	**91.0 (86.4–94.5)**	**53.2 (42.6–63.6)**
**Sex**						
Male	219	85.1 (78.6–90.2)	93.1 (83.3–98.1)	141	91.2 (83.4–96.1)	56.0 (41.3–70.0)
Female	240	83.6 (77.6–88.5)	95.6 (84.9–99.5)	165	90.9 (84.3–95.4)	50.0 (34.6–65.4)
**Age group**						
18–30 years	74	87.7 (76.3–94.9)	94.1 (71.3–99.9)	51	82.1 (66.5–92.5)	58.2 (27.7–84.8)
31–40 years	142	86.6 (79.6–91.8)	87.5 (47.3–99.7)	117	93.8 (86.0–97.9)	62.2 (44.8–77.5)
41–50 years	113	85.4 (76.7–91.8)	100 (80.5–100)	82	93.1 (83.3–98.1)	41.7 (22.1–63.4)
51–60 years	87	75.0 (61.1–86.0)	100 (90.0–100)	39	92.0 (74.0–99.0)	42.9 (17.7–71.1)
>60 years	43	76.5 (50.1–93.2)	84.6 (65.1–95.6)	17	90.0 (55.5–99.7)	57.1 (18.4–90.1)
**Area of enrolment/hospitalisation**						
Urban	330	85.4 (80.4–89.5)	98.7 (93.0–100)	217	92.3 (86.9–96.0)	55.7 (42.4–68.5)
Rural	129	81.6 (72.7–88.5)	80.8 (60.6–93.4)	89	87.5 (75.9–94.8)	48.5 (30.8–66.5)
**Site of death**						
Hospital or other health facility	351	85.0 (80.0–89.1)	96.9 (91.3–99.4)	218	91.1 (85.6–95.1)	55.0 (41.6–67.9)
Home or unknown location‡	108	82.5 (73.8–89.3)	40.0 (5.3–85.3)	88	90.7 (79.7–96.9)	50.0 (32.4–67.6)
**Time from death to VA**						
≤90 days	88	93.3 (85.1–97.8)	100 (75.3–100)	70	92.0 (80.8–97.8)	45.0 (23.1–68.5)
91–180 days	111	88.4 (78.4–94.9)	100 (91.6–100)	61	94.2 (84.1–98.8)	44.4 (13.7–78.8)
181–270 days	90	83.8 (72.9–91.6)	100 (84.6–100)	57	90.5 (77.4–97.3)	53.3 (26.6–78.7)
>270 days	170	77.8 (70.1–84.3)	76.9 (56.4–91.0)	118	88.2 (78.1–94.8)	58.0 (43.2–71.8)

* "Was there any diagnosis of HIV/AIDS?"

† "Did s/he ever take ART?"

‡ Estimates of specificity for those dying at home/in the community are limited because 98/103 (96%) HIV-negative individuals died in hospitals

ART: antiretroviral therapy; CI: confidence interval; VA: verbal autopsy

Supplementary table 3. Specificity of VA methods in assigning HIV-associated causes of death compared with confirmed serostatus, stratified by sex, age group, area of enrolment/hospitalisation, site of death, and time from death to VA (n = 459)

Group/sub-group	N	Specificity of VA methods compared with confirmed serostatus, % (95 CI)				
		**PCVA**	**InterVA-4***	**InterVA-5***	**SVA v1.1.1**	**SVA v1.2.1**
**Overall**	**459**	**96.1 (90.4–98.9)**	**89.3 (81.7–94.5)**	**87.4 (79.4–93.1)**	**95.1 (89.0–98.4)**	**95.1 (89.0–98.4)**
**Sex**						
Male	219	94.8 (85.6–98.9)	89.7 (78.8–96.1)	87.9 (76.7–95.0)	93.1 (83.3–98.1)	93.1 (83.3–98.1)
Female	240	97.8 (88.2–99.9)	88.9 (75.9–96.3)	86.7 (73.2–94.9)	97.8 (88.2–99.9)	97.8 (88.2–99.9)
**Age group**						
18–30 years	74	88.2 (63.6–98.5)	76.5 (50.1–93.2)	64.7 (38.3–85.8)	100 (80.5–100)	94.1 (71.3–99.9)
31–40 years	142	87.5 (47.3–99.7)	87.5 (47.3–99.7)	75.0 (34.9–96.8)	75.0 (34.9–96.8)	87.5 (47.3–99.7)
41–50 years	113	100 (80.5–100)	94.1 (71.3–99.9)	94.1 (71.3–99.9)	88.2 (63.6–98.5)	100 (80.5–100)
51–60 years	87	100 (90.0–100)	91.4 (76.9–98.2)	94.3 (80.8–99.3)	97.1 (85.1–99.9)	100 (90.0–100)
>60 years	43	96.2 (80.4–99.9)	92.3 (74.9–99.1)	92.3 (74.9–99.1)	100 (86.8–100)	88.5 (69.8–97.6)
**Area of enrolment/hospitalisation**						
Urban	330	98.7 (93.0–100)	93.5 (85.5–97.9)	90.9 (82.2–96.3)	97.4 (90.9–99.7)	98.7 (93.0–100)
Rural	129	88.5 (69.8–97.6)	76.9 (56.4–91.0)	76.9 (56.4–91.0)	88.5 (69.8–97.6)	84.6 (65.1–95.6)
**Site of death**						
Hospital or other health facility	351	99.0 (94.4–100)	89.8 (82.0–95.0)	89.8 (82.0–95.0)	95.9 (89.9–98.9)	98.0 (92.8–99.8)
Home or unknown location†	108	40.0 (5.3–85.3)	80.0 (28.4–99.5)	40.0 (5.3–85.3)	80.0 (28.4–99.5)	40.0 (5.3–85.3)
**Time from death to VA**						
≤90 days	88	100 (75.3–100)	100 (75.3–100)	100 (75.3–100)	100 (75.3–100)	100 (75.3–100)
91–180 days	111	100 (91.6–100)	83.3 (68.6–93.0)	85.7 (71.5–94.6)	95.2 (83.8–99.4)	100 (91.6–100)
181–270 days	90	95.5 (77.2–99.9)	90.9 (70.8–98.9)	90.9 (70.8–98.9)	95.5 (77.2–99.9)	100 (84.6–100)
>270 days	170	88.5 (69.8–97.6)	92.3 (74.9–99.1)	80.8 (60.6–93.4)	92.3 (74.9–99.1)	80.8 (60.6–93.4)

* Individuals considered 'test positive' if HIV/AIDS assigned as most likely CoD

† Estimates of specificity for those dying at home/in the community are limited because 98/103 (96%) HIV-negative individuals died in hospitals

CI: confidence interval; PCVA: physician-certified verbal autopsy; SVA: SmartVA-Analyze; VA: verbal autopsy

Supplementary table 4. Specificity of VA methods in assigning HIV-associated causes of death compared with VA-reported HIV status, stratified by sex, age group, area of enrolment/hospitalisation, site of death, and time from death to VA (n = 459)

Group/sub-group	N	Specificity of VA methods compared with VA-reported HIV status, % (95 CI)				
		PCVA	InterVA-4*	InterVA-5*	SVA v1.1.1	SVA v1.2.1
**Overall**	**459**	81.0 (73.9–86.9)	83.0 (76.1–88.6)	78.4 (71.1–84.7)	90.2 (84.3–94.4)	97.4 (93.4–99.3)
**Sex**						
Male	219	85.9 (76.2–92.7)	92.3 (84.0–97.1)	85.9 (76.2–92.7)	89.7 (80.8–95.5)	98.7 (93.1–100)
Female	240	76.0 (64.7–85.1)	73.3 (61.9–82.9)	70.7 (59.0–80.6)	90.7 (81.7–96.2)	96.0 (88.8–99.2)
**Age group**						
18–30 years	74	78.3 (56.3–92.5)	69.6 (47.1–86.8)	60.9 (38.5–80.3)	91.3 (72.0–98.9)	100 (85.2–100)
31–40 years	142	44.0 (24.4–65.1)	76.0 (54.9–90.6)	64.0 (42.5–82.0)	76.0 (54.9–90.6)	92.0 (74.0–99)
41–50 years	113	77.4 (58.9–90.4)	83.9 (66.3–94.5)	77.4 (58.9–90.4)	80.6 (62.5–92.5)	96.8 (83.3–99.9)
51–60 years	87	93.8 (82.8–98.7)	85.4 (72.2–93.9)	85.4 (72.2–93.9)	97.9 (88.9–99.9)	97.9 (88.9–99.9)
>60 years	43	100 (86.8–100)	96.2 (80.4–99.9)	96.2 (80.4–99.9)	100 (86.8–100)	100 (86.8–100)
**Area of enrolment/hospitalisation**						
Urban	330	81.4 (73.0–88.1)	88.5 (81.1–93.7)	82.3 (74.0–88.8)	91.2 (84.3–95.7)	98.2 (93.8–99.8)
Rural	129	80.0 (64.4–90.9)	67.5 (50.9–81.4)	67.5 (50.9–81.4)	87.5 (73.2–95.8)	95.0 (83.1–99.4)
**Site of death**						
Hospital or other health facility	351	85.7 (78.6–91.2)	83.5 (76.0–89.3)	80.5 (72.7–86.8)	91.0 (84.8–95.3)	97.0 (92.5–99.2)
Home or unknown location†	108	50.0 (27.2–72.8)	50.0 (27.2–72.8)	65.0 (40.8–84.6)	85.0 (62.1–96.8)	100 (83.2–100)
**Time from death to VA**						
≤90 days	88	88.9 (65.3–98.6)	88.9 (65.3–98.6)	88.9 (65.3–98.6)	100 (81.5–100)	100 (81.5–100)
91–180 days	111	92.0 (80.8–97.8)	84.0 (70.9–92.8)	84.0 (70.9–92.8)	92.0 (80.8–97.8)	100 (92.9–100)
181–270 days	90	75.8 (57.7–88.9)	81.8 (64.5–93.0)	75.8 (57.7–88.9)	90.9 (75.7–98.1)	93.9 (79.8–99.3)
>270 days	170	71.2 (56.9–82.9)	80.8 (67.5–90.4)	71.2 (56.9–82.9)	84.6 (71.9–93.1)	96.2 (86.8–99.5)

* Individuals considered 'test positive' if HIV/AIDS assigned as most likely CoD

† Estimates of specificity for those dying at home/in the community are limited because 98/103 (96%) HIV-negative individuals died in hospitals

CI: confidence interval; PCVA: physician-certified verbal autopsy; SVA: SmartVA-Analyze; VA: verbal autopsy

Supplementary table 5. Immediate and underlying causes of death assigned by PCVA prior to processing by MMDS; causes of death assigned by InterVA-4 and InterVA-5 (with associated likelihoods) prior to calculation of CSMFs; and causes of death assigned by SmartVA-Analyze v1.1.1 and v1.2.1 for all decedents (n = 459)

Participant details				VA	PCVA				InterVA-4						InterVA-5						SmartVA-Analyze v1.1.1		SmartVA-Analyze v.1.2.1	
ID	Sex	Age*	HIV status	HIV status	iCoD	uCoD	MMDS CoD	HIV CoD ?	Cause 1	Lik 1	Cause 2	Lik 2	Cause 3	Lik 3	Cause 1	Lik 1	Cause 2	Lik 2	Cause 3	Lik 3	CoD	HIV CoD ?	CoD	HIV CoD ?
1	F	24.3	Pos	Pos	A19	B20	B20	Yes	HIV/AIDS	100					HIV/AIDS	99					Breast Cancer	No	AIDS	Yes
2	M	45.3	Pos	Pos	A16	B20	B20	Yes	HIV/AIDS	76					HIV/AIDS	99					AIDS	Yes	AIDS	Yes
3	M	33.1	Pos	Pos	A16	B20	B20	Yes	Malaria	56	ARTI	44			HIV/AIDS	88					AIDS	Yes	AIDS	Yes
4	F	61.6	Pos	Pos	K76	B24	B23	Yes	HIV/AIDS	100					HIV/AIDS	99					Diabetes	No	AIDS	Yes
5	F	23.1	Pos	DK	B24		B24	Yes	HIV/AIDS	100					HIV/AIDS	99					AIDS	Yes	Undetermined	No
6	F	26.9	Pos	Pos	N94	B24	B24	Yes	HIV/AIDS	99					HIV/AIDS	99					Breast Cancer	No	AIDS	Yes
7	F	41.0	Pos	DK	B59	B20	B20	Yes	HIV/AIDS	100					HIV/AIDS	99					Breast Cancer	No	Undetermined	No
8	M	53.5	Pos	Pos	A16	B20	B20	Yes	HIV/AIDS	100					HIV/AIDS	99					Undetermined	No	AIDS	Yes
9	F	33.3	Pos	Pos	A17	B20	B20	Yes	HIV/AIDS	100					HIV/AIDS	99					Leukemia/Lymphomas	No	Leukemia/Lymphomas	No
10	M	28.4	Pos	Pos	K71	B20	K71	No	Pulmonary TB	80					HIV/AIDS	98					Undetermined	No	AIDS	Yes
11	M	38.7	Pos	Pos	A15	B20	B20	Yes	HIV/AIDS	76					HIV/AIDS	99					Diabetes	No	AIDS	Yes
12	M	57.6	Pos	Pos	A16	B20	B20	Yes	HIV/AIDS	100					HIV/AIDS	99					Leukemia/Lymphomas	No	AIDS	Yes
13	F	30.9	Pos	Pos	N17	A15	B20	Yes	ARTI	100					ARTI	82					Undetermined	No	AIDS	Yes
14	F	32.7	Pos	Pos	A16	B20	B20	Yes	HIV/AIDS	100					HIV/AIDS	99					Diarrhea/Dysentery	No	AIDS	Yes
15	M	37.3	Pos	Pos	A18	B20	B20	Yes	HIV/AIDS	71					HIV/AIDS	85					AIDS	Yes	AIDS	Yes
16	M	40.3	Pos	Pos	A16	B20	B20	Yes	Pulmonary TB	97					HIV/AIDS	98					AIDS	Yes	AIDS	Yes
17	M	42.8	Pos	DK	T09	V89	X59	No	Pulmonary TB	99					Pulmonary TB	40	Other Ca	39			Road Traffic	No	TB	No
18	F	22.2	Pos	Pos	A16	B24	B20	Yes	HIV/AIDS	100					HIV/AIDS	99					Pneumonia	No	AIDS	Yes
19	F	40.7	Pos	Neg	A09	B24	A09	No	Stroke	100					Stroke	99					Undetermined	No	Stroke	No
20	F	39.5	Pos	Pos	A15	B20	B20	Yes	Pregnancy-induced hypertension	45	Acute cardiac dx	23			Other cardiac dx	90					Undetermined	No	AIDS	Yes
21	F	48.8	Pos	Pos	B24		B24	Yes	Diarrhoeal dx	90					Diarrhoeal dx	95					Breast Cancer	No	AIDS	Yes

Participant details				VA	PCVA				InterVA-4						InterVA-5						SmartVA-Analyze v1.1.1		SmartVA-Analyze v.1.2.1	
ID	Sex	Age*	HIV status	HIV status	iCoD	uCoD	MMDS CoD	HIV CoD?	Cause 1	Lik 1	Cause 2	Lik 2	Cause 3	Lik 3	Cause 1	Lik 1	Cause 2	Lik 2	Cause 3	Lik 3	CoD	HIV CoD?	CoD	HIV CoD?
22	F	48.4	Pos	Pos	A16	B20	B20	Yes	Acute abdomen	77					HIV/AIDS	99					Leukemia/Lymphomas	No	AIDS	Yes
23	F	43.1	Pos	Pos	G40	B24	G40	No	HIV/AIDS	100					HIV/AIDS	99					AIDS	Yes	AIDS	Yes
24	F	45.1	Pos	Pos	A16	B20	B20	Yes	HIV/AIDS	100					HIV/AIDS	99					AIDS	Yes	AIDS	Yes
25	F	42.5	Pos	Pos	D64	B23	B23	Yes	HIV/AIDS	98					HIV/AIDS	85					Undetermined	No	AIDS	Yes
26	M	42.8	Pos	Pos	N19	B24	B23	Yes	HIV/AIDS	100					HIV/AIDS	99					Leukemia/Lymphomas	No	AIDS	Yes
27	M	36.0	Pos	DK	A16	B20	B20	Yes	ARTI	97					ARTI	99					Undetermined	No	Undetermined	No
28	F	34.5	Pos	Pos	B24		B24	Yes	HIV/AIDS	100					HIV/AIDS	99					Leukemia/Lymphomas	No	AIDS	Yes
29	M	41.0	Pos	Pos	K72	A16	B20	Yes	Pulmonary TB	100					Pulmonary TB	99					TB	No	AIDS	Yes
30	M	33.1	Pos	Pos	K71	A16	K71	No	HIV/AIDS	100					HIV/AIDS	99					AIDS	Yes	AIDS	Yes
31	F	32.3	Pos	Pos	B20		B20	Yes	HIV/AIDS	100					HIV/AIDS	99					Breast Cancer	No	AIDS	Yes
32	F	45.6	Pos	Pos	A16	B20	B20	Yes	HIV/AIDS	100					HIV/AIDS	99					Asthma	No	AIDS	Yes
33	M	33.9	Pos	Pos	A15	B20	B20	Yes	HIV/AIDS	100					HIV/AIDS	99					AIDS	Yes	AIDS	Yes
34	M	65.0	Pos	Pos	I74	E11	I82	No	HIV/AIDS	98					HIV/AIDS	99					AIDS	Yes	AIDS	Yes
35	F	34.8	Pos	Pos	G03	B20	B20	Yes	HIV/AIDS	100					HIV/AIDS	99					Diabetes	No	AIDS	Yes
36	M	35.1	Pos	DK	A19	B20	B20	Yes	Digestive Ca	98					Digestive Ca	88					Diarrhea/Dysentery	No	TB	No
37	F	25.9	Pos	Pos	A16	B20	B20	Yes	Acute abdomen	99					Acute abdomen	95					Breast Cancer	No	AIDS	Yes
38	M	36.8	Pos	Pos	K92	B24	B23	Yes	Digestive Ca	100					Digestive Ca	99					Undetermined	No	AIDS	Yes
39	F	39.3	Pos	Pos	A17	B20	B20	Yes	HIV/AIDS	100					HIV/AIDS	99					Diarrhea/Dysentery	No	AIDS	Yes
40	M	51.7	Pos	Pos	B24		B24	Yes	Other NCD	100					Indeterminate	0					Lung Cancer	No	AIDS	Yes
41	F	31.4	Pos	Pos	C53	B21	C53	No	HIV/AIDS	93					HIV/AIDS	79					Stomach Cancer	No	AIDS	Yes
42	F	23.1	Pos	Pos	A19	B20	B20	Yes	HIV/AIDS	100					HIV/AIDS	99					Leukemia/Lymphomas	No	AIDS	Yes
43	F	24.9	Pos	Pos	I64	B20	B23	Yes	Meningitis/encephalitis	100					Meningitis/encephalitis	99					Undetermined	No	AIDS	Yes
44	M	41.1	Pos	Pos	B24		B24	Yes	HIV/AIDS	94					HIV/AIDS	99					AIDS	Yes	AIDS	Yes

Participant details				VA	PCVA				InterVA-4						InterVA-5						SmartVA-Analyze v1.1.1		SmartVA-Analyze v.1.2.1	
ID	Sex	Age*	HIV status	HIV status	iCoD	uCoD	MMDS CoD	HIV CoD?	Cause 1	Lik 1	Cause 2	Lik 2	Cause 3	Lik 3	Cause 1	Lik 1	Cause 2	Lik 2	Cause 3	Lik 3	CoD	HIV CoD?	CoD	HIV CoD?
45	M	41.3	Pos	DK	B24		B24	Yes	Pulmonary TB	100					HIV/AIDS	54	Pulmonary TB	44			AIDS	Yes	TB	No
46	F	32.9	Pos	Pos	A17	B20	B20	Yes	Pulmonary TB	86					HIV/AIDS	99					AIDS	Yes	AIDS	Yes
47	M	42.2	Pos	Pos	A15	B20	B20	Yes	Pulmonary TB	57	HIV/AIDS	35			HIV/AIDS	99					AIDS	Yes	AIDS	Yes
48	M	45.1	Pos	Pos	I64	B23	B23	Yes	HIV/AIDS	100					HIV/AIDS	99					Pneumonia	No	AIDS	Yes
49	F	33.0	Pos	Pos	B20		B20	Yes	HIV/AIDS	100					HIV/AIDS	99					Diarrhea/Dysentery	No	AIDS	Yes
50	M	39.4	Pos	Pos	A18	B20	B20	Yes	HIV/AIDS	88					HIV/AIDS	99					AIDS	Yes	AIDS	Yes
51	F	47.3	Pos	Pos	G03	B20	B20	Yes	HIV/AIDS	100					HIV/AIDS	99					AIDS	Yes	Breast Cancer	No
52	M	61.6	Pos	Pos	N19	B24	B23	Yes	Pulmonary TB	56	ARTI	44			HIV/AIDS	99					Undetermined	No	AIDS	Yes
53	F	34.4	Pos	Pos	A09	B20	A09	No	HIV/AIDS	99					HIV/AIDS	99					Diarrhea/Dysentery	No	AIDS	Yes
54	F	42.6	Pos	Pos	K27	B23	B23	Yes	HIV/AIDS	97					HIV/AIDS	79					AIDS	Yes	AIDS	Yes
55	M	40.1	Pos	Pos	A15	B20	B20	Yes	HIV/AIDS	42	Digestive Ca	41			HIV/AIDS	98					Diarrhea/Dysentery	No	AIDS	Yes
56	M	31.8	Pos	Pos	A15	B20	B20	Yes	Pulmonary TB	100					Pulmonary TB	99					Undetermined	No	AIDS	Yes
57	M	42.0	Pos	Pos	G00	B20	B20	Yes	Digestive Ca	99					Digestive Ca	87					AIDS	Yes	AIDS	Yes
58	F	23.2	Pos	Pos	A16	B20	B20	Yes	ARTI	94					ARTI	88					AIDS	Yes	AIDS	Yes
59	F	40.5	Pos	Pos	A16	B20	B20	Yes	HIV/AIDS	100					HIV/AIDS	99					AIDS	Yes	AIDS	Yes
60	M	57.4	Pos	Pos	A19	B20	B20	Yes	Digestive Ca	100					Digestive Ca	99					Diarrhea/Dysentery	No	AIDS	Yes
61	M	49.2	Pos	Pos	A16	B20	B20	Yes	HIV/AIDS	79					HIV/AIDS	99					Diabetes	No	AIDS	Yes
62	F	44.9	Pos	Pos	A16	B20	B20	Yes	Pulmonary TB	100					Pulmonary TB	83					AIDS	Yes	AIDS	Yes
63	F	48.7	Pos	Pos	A17	B20	B20	Yes	HIV/AIDS	100					HIV/AIDS	99					Undetermined	No	AIDS	Yes
64	M	40.4	Pos	Pos	A16	B20	B20	Yes	Reproductive Ca	65					Renal failure	88					Prostate Cancer	No	AIDS	Yes
65	F	21.4	Pos	Neg	K71	A16	B20	Yes	Pulmonary TB	82					HIV/AIDS	93					AIDS	Yes	TB	No
66	M	53.8	Pos	Pos	A48	B24	B20	Yes	Digestive Ca	93					Digestive Ca	97					Diarrhea/Dysentery	No	AIDS	Yes
67	F	30.2	Pos	Pos	B24		B24	Yes	HIV/AIDS	100					HIV/AIDS	99					Diarrhea/Dysentery	No	AIDS	Yes

Participant details				VA	PCVA				InterVA-4						InterVA-5						SmartVA-Analyze v1.1.1		SmartVA-Analyze v.1.2.1	
ID	Sex	Age*	HIV status	HIV status	iCoD	uCoD	MMDS CoD	HIV CoD ?	Cause 1	Lik 1	Cause 2	Lik 2	Cause 3	Lik 3	Cause 1	Lik 1	Cause 2	Lik 2	Cause 3	Lik 3	CoD	HIV CoD ?	CoD	HIV CoD ?
68	F	24.7	Pos	Pos	K76	A16	B20	Yes	HIV/AIDS	100					HIV/AIDS	99					Undetermined	No	AIDS	Yes
69	M	44.3	Pos	Pos	D64	B20	B20	Yes	Acute abdomen	100					Acute abdomen	96					Undetermined	No	AIDS	Yes
70	F	33.5	Pos	Pos	A15	B20	B20	Yes	HIV/AIDS	100					HIV/AIDS	99					Breast Cancer	No	AIDS	Yes
71	M	29.3	Pos	Pos	A17	B20	B20	Yes	ARTI	86					HIV/AIDS	99					TB	No	AIDS	Yes
72	F	32.9	Pos	Pos	K27	B24	B23	Yes	HIV/AIDS	97					HIV/AIDS	96					Stomach Cancer	No	AIDS	Yes
73	M	63.1	Pos	Pos	A16	B20	B20	Yes	Pulmonary TB	65					HIV/AIDS	83					Diabetes	No	AIDS	Yes
74	F	35.9	Pos	Pos	A19	B20	B20	Yes	Digestive Ca	92					HIV/AIDS	86					Diarrhea/Dysentery	No	AIDS	Yes
75	M	36.8	Pos	Pos	A15	B20	B20	Yes	Pulmonary TB	100					Pulmonary TB	96					Undetermined	No	AIDS	Yes
76	F	30.8	Pos	Pos	A16	B20	B20	Yes	HIV/AIDS	61	Pulmonary TB	39			HIV/AIDS	99					Breast Cancer	No	AIDS	Yes
77	M	31.6	Pos	Pos	A09	B20	A09	No	HIV/AIDS	97					HIV/AIDS	95					Undetermined	No	AIDS	Yes
78	M	37.5	Pos	Pos	A09	B20	A09	No	Digestive Ca	100					Digestive Ca	99					Other Non-communicable Diseases	No	Other Non-communicable Diseases	No
79	M	26.0	Pos	Pos	A16	B20	B20	Yes	Diabetes	81					Diabetes	99					Undetermined	No	AIDS	Yes
80	M	31.8	Pos	Pos	A16	B20	B20	Yes	HIV/AIDS	100					HIV/AIDS	99					TB	No	AIDS	Yes
81	F	39.0	Pos	DK	B24		B24	Yes	Digestive Ca	52	Respiratory Ca	33			Digestive Ca	86					Breast Cancer	No	Undetermined	No
82	F	32.5	Pos	Pos	B24		B24	Yes	HIV/AIDS	100					HIV/AIDS	99					Diarrhea/Dysentery	No	AIDS	Yes
83	F	37.9	Pos	Pos	I64	B23	B23	Yes	Stroke	100					Stroke	99					Breast Cancer	No	Stroke	No
84	M	30.1	Pos	Pos	A16	B20	B20	Yes	Digestive Ca	70					Digestive Ca	75					Other Non-communicable Diseases	No	AIDS	Yes
85	M	43.5	Pos	Pos	B24		B24	Yes	Pulmonary TB	100					Pulmonary TB	99					TB	No	AIDS	Yes
86	F	58.0	Pos	Pos	G40	B24	G40	No	HIV/AIDS	100					HIV/AIDS	99					Epilepsy	No	AIDS	Yes
87	F	35.1	Pos	Pos	R11	B24	B24	Yes	HIV/AIDS	100					HIV/AIDS	99					AIDS	Yes	AIDS	Yes
88	F	34.0	Pos	Pos	N17	B24	B23	Yes	HIV/AIDS	99					HIV/AIDS	67					AIDS	Yes	AIDS	Yes
89	M	48.0	Pos	Pos	B24		B24	Yes	Pulmonary TB	56	ARTI	44			ARTI	83					AIDS	Yes	AIDS	Yes
90	M	39.1	Pos	Pos	A16	B20	B20	Yes	HIV/AIDS	95					HIV/AIDS	99					AIDS	Yes	AIDS	Yes

Participant details				VA	PCVA				InterVA-4						InterVA-5						SmartVA-Analyze v1.1.1		SmartVA-Analyze v.1.2.1	
ID	Sex	Age*	HIV status	HIV status	iCoD	uCoD	MMDS CoD	HIV CoD?	Cause 1	Lik 1	Cause 2	Lik 2	Cause 3	Lik 3	Cause 1	Lik 1	Cause 2	Lik 2	Cause 3	Lik 3	CoD	HIV CoD?	CoD	HIV CoD?
91	M	47.6	Pos	Pos	A15	B20	B20	Yes	Pulmonary TB	100					Pulmonary TB	99					AIDS	Yes	AIDS	Yes
92	F	50.3	Pos	Pos	B45	B20	B20	Yes	Digestive Ca	100					Digestive Ca	99					Diarrhea/Dysentery	No	AIDS	Yes
93	F	28.7	Pos	Pos	B24		B24	Yes	HIV/AIDS	100					HIV/AIDS	99					Breast Cancer	No	Breast Cancer	No
94	M	36.5	Pos	Pos	A19	B20	B20	Yes	HIV/AIDS	100					HIV/AIDS	99					AIDS	Yes	AIDS	Yes
95	F	36.0	Pos	Pos	A16	B20	B20	Yes	Severe anaemia	85					HIV/AIDS	81					Undetermined	No	AIDS	Yes
96	F	34.5	Pos	Pos	A16	B20	B20	Yes	Pulmonary TB	100					Pulmonary TB	99					Breast Cancer	No	AIDS	Yes
97	M	37.4	Pos	Pos	A09	B20	A09	No	HIV/AIDS	100					HIV/AIDS	99					Leukemia/Lymphomas	No	AIDS	Yes
98	M	38.9	Pos	Pos	A15	B20	B20	Yes	Pulmonary TB	93					Other Ca	75					Asthma	No	AIDS	Yes
99	F	22.5	Pos	Pos	A19	B20	B20	Yes	HIV/AIDS	100					HIV/AIDS	99					AIDS	Yes	AIDS	Yes
100	M	45.9	Pos	Pos	B24		B24	Yes	Digestive Ca	99					Digestive Ca	99					Diarrhea/Dysentery	No	AIDS	Yes
101	M	27.0	Pos	Pos	T37	B24	X44	No	Pulmonary TB	98					HIV/AIDS	49	Pulmonary TB	49			Pneumonia	No	AIDS	Yes
102	F	39.3	Pos	Pos	A16	B20	B20	Yes	HIV/AIDS	100					HIV/AIDS	99					Diabetes	No	AIDS	Yes
103	F	48.2	Pos	Pos	K71	A16	B20	Yes	HIV/AIDS	95					HIV/AIDS	99					Breast Cancer	No	AIDS	Yes
104	F	27.0	Pos	Pos	A09	B20	A09	No	ARTI	95					HIV/AIDS	86					AIDS	Yes	AIDS	Yes
105	F	36.7	Pos	Pos	K70	B24	K70	No	Digestive Ca	71					Digestive Ca	93					AIDS	Yes	AIDS	Yes
106	F	41.5	Pos	Pos	K92	B20	B23	Yes	HIV/AIDS	100					HIV/AIDS	99					Diarrhea/Dysentery	No	AIDS	Yes
107	M	49.7	Pos	Pos	N18	B24	B23	Yes	HIV/AIDS	100					Other Ca	96					AIDS	Yes	AIDS	Yes
108	M	64.8	Pos	Pos	A16	B20	B20	Yes	ARTI	100					ARTI	99					Undetermined	No	AIDS	Yes
109	F	63.8	Pos	Pos	B59	B20	B20	Yes	Pulmonary TB	97					Pulmonary TB	76					Undetermined	No	AIDS	Yes
110	F	41.9	Pos	Pos	C50	B21	C50	No	Digestive Ca	100					Digestive Ca	99					Breast Cancer	No	AIDS	Yes
111	F	41.9	Pos	Pos	A15	B20	B20	Yes	Pulmonary TB	41	HIV/AIDS	33	ARTI	26	HIV/AIDS	99					Leukemia/Lymphomas	No	AIDS	Yes
112	F	32.3	Pos	Pos	J15	B20	B20	Yes	HIV/AIDS	82					HIV/AIDS	99					AIDS	Yes	AIDS	Yes
113	F	24.9	Pos	Pos	B45	B20	B20	Yes	HIV/AIDS	100					HIV/AIDS	99					Breast Cancer	No	AIDS	Yes
114	M	45.9	Pos	Pos	I64	B20	B23	Yes	Cirrhosis	97					Cirrhosis	67					Undetermined	No	AIDS	Yes

Participant details				VA	PCVA				InterVA-4						InterVA-5						SmartVA-Analyze v1.1.1		SmartVA-Analyze v.1.2.1	
ID	Sex	Age*	HIV status	HIV status	iCoD	uCoD	MMDS CoD	HIV CoD ?	Cause 1	Lik 1	Cause 2	Lik 2	Cause 3	Lik 3	Cause 1	Lik 1	Cause 2	Lik 2	Cause 3	Lik 3	CoD	HIV CoD ?	CoD	HIV CoD ?
115	M	38.8	Pos	Pos	A15	B20	B20	Yes	Pulmonary TB	51	Digestive Ca	41			Digestive Ca	79					Road Traffic	No	AIDS	Yes
116	F	36.7	Pos	Pos	A16	B20	B20	Yes	HIV/AIDS	99					HIV/AIDS	99					Diabetes	No	AIDS	Yes
117	F	26.4	Pos	Pos	D64	B20	B20	Yes	HIV/AIDS	100					HIV/AIDS	99					Breast Cancer	No	Breast Cancer	No
118	F	62.1	Pos	Pos	I64	B24	B23	Yes	Stroke	63					Stroke	92					Stroke	No	AIDS	Yes
119	M	49.0	Pos	DK	I50	I10	I50	No	Diabetes	99					Diabetes	99					Undetermined	No	TB	No
120	M	55.6	Pos	Pos	A16	B20	B20	Yes	Pulmonary TB	93					HIV/AIDS	99					Undetermined	No	AIDS	Yes
121	M	55.6	Pos	Neg	X72		W32	No	Assault	100					Assault	71					Suicide	No	Road Traffic	No
122	F	34.0	Pos	Pos	B59	B20	B20	Yes	HIV/AIDS	96					HIV/AIDS	99					Breast Cancer	No	AIDS	Yes
123	F	57.0	Pos	Pos	A15	B20	B20	Yes	Pulmonary TB	86					Pulmonary TB	92					AIDS	Yes	AIDS	Yes
124	M	51.8	Pos	Pos	B24		B24	Yes	HIV/AIDS	100					HIV/AIDS	99					Undetermined	No	AIDS	Yes
125	M	34.6	Pos	Pos	K72	A15	B20	Yes	Digestive Ca	100					Digestive Ca	60	HIV/AIDS	38			Diarrhea/Dysentery	No	AIDS	Yes
126	F	49.1	Pos	Pos	A16	B24	B20	Yes	HIV/AIDS	100					HIV/AIDS	99					Diarrhea/Dysentery	No	AIDS	Yes
127	M	43.8	Pos	Pos	K76	B24	B23	Yes	HIV/AIDS	95					HIV/AIDS	99					Undetermined	No	AIDS	Yes
128	M	36.2	Pos	DK	A15	B20	B20	Yes	Pulmonary TB	95					HIV/AIDS	99					AIDS	Yes	Undetermined	No
129	M	47.6	Pos	Pos	G00	B20	B20	Yes	HIV/AIDS	100					HIV/AIDS	99					Other Non-communicable Diseases	No	AIDS	Yes
130	M	41.5	Pos	Pos	A16	B20	B20	Yes	Stroke	84					Stroke	59					AIDS	Yes	AIDS	Yes
131	F	55.5	Pos	Pos	A16	B20	B20	Yes	Diarrhoeal dx	75					Diarrhoeal dx	67					Undetermined	No	AIDS	Yes
132	M	28.2	Pos	DK	A19	B20	B20	Yes	Pulmonary TB	100					Pulmonary TB	98					Undetermined	No	TB	No
133	F	56.2	Pos	Pos	A09	B20	A09	No	Digestive Ca	98					Digestive Ca	98					Undetermined	No	AIDS	Yes
134	F	33.8	Pos	Pos	A16	B20	B20	Yes	HIV/AIDS	100					HIV/AIDS	99					Diarrhea/Dysentery	No	AIDS	Yes
135	F	62.4	Pos	Neg	G40		G40	No	Renal failure	96					Renal failure	95					Undetermined	No	Undetermined	No
136	F	27.4	Pos	Pos	B22		B24	Yes	HIV/AIDS	100					HIV/AIDS	99					Stomach Cancer	No	AIDS	Yes
137	M	45.1	Pos	Pos	A15	B20	B20	Yes	HIV/AIDS	100					HIV/AIDS	99					TB	No	AIDS	Yes

Participant details				VA	PCVA				InterVA-4						InterVA-5						SmartVA-Analyze v1.1.1		SmartVA-Analyze v.1.2.1	
ID	Sex	Age*	HIV status	HIV status	iCoD	uCoD	MMDS CoD	HIV CoD ?	Cause 1	Lik 1	Cause 2	Lik 2	Cause 3	Lik 3	Cause 1	Lik 1	Cause 2	Lik 2	Cause 3	Lik 3	CoD	HIV CoD ?	CoD	HIV CoD ?
138	F	52.6	Pos	Pos	A16	B20	B20	Yes	Digestive Ca	81					HIV/AIDS	95					Leukemia/Lymphomas	No	AIDS	Yes
139	F	30.6	Pos	Neg	A19	B20	B20	Yes	Digestive Ca	99					Digestive Ca	94					Diabetes	No	Diabetes	No
140	M	32.9	Pos	Pos	J15	B20	B20	Yes	ARTI	100					ARTI	99					Asthma	No	AIDS	Yes
141	F	35.8	Pos	Pos	B24		B24	Yes	Diarrhoeal dx	60					Diarrhoeal dx	65					Breast Cancer	No	AIDS	Yes
142	F	32.5	Pos	Pos	G03	B20	B20	Yes	HIV/AIDS	100					HIV/AIDS	99					Undetermined	No	AIDS	Yes
143	M	32.9	Pos	Pos	B24		B24	Yes	Reproductive Ca	81					Digestive Ca	49					Leukemia/Lymphomas	No	AIDS	Yes
144	F	36.6	Pos	Pos	B24		B24	Yes	Diarrhoeal dx	99					Diarrhoeal dx	92					Undetermined	No	AIDS	Yes
145	F	36.7	Pos	Pos	B24		B24	Yes	HIV/AIDS	100					HIV/AIDS	99					AIDS	Yes	AIDS	Yes
146	M	31.6	Pos	Pos	Q20	B24	Q24	No	Acute abdomen	73					Diabetes	66					Cirrhosis	No	AIDS	Yes
147	M	29.9	Pos	Pos	B24		B24	Yes	HIV/AIDS	100					HIV/AIDS	99					Diabetes	No	AIDS	Yes
148	F	22.7	Pos	Pos	A17	B20	B20	Yes	Acute abdomen	97					HIV/AIDS	88					Undetermined	No	AIDS	Yes
149	M	31.1	Pos	Pos	B24		B24	Yes	HIV/AIDS	100					HIV/AIDS	99					Diabetes	No	Diabetes	No
150	F	32.6	Pos	Pos	A16	B20	B20	Yes	HIV/AIDS	100					HIV/AIDS	99					AIDS	Yes	AIDS	Yes
151	F	39.2	Pos	Pos	A16	B20	B20	Yes	Cirrhosis	96					Cirrhosis	71					AIDS	Yes	AIDS	Yes
152	M	40.2	Pos	Pos	G00	K92	B23	Yes	HIV/AIDS	99					HIV/AIDS	96					AIDS	Yes	AIDS	Yes
153	M	33.2	Pos	Pos	A09	B20	A09	No	HIV/AIDS	100					HIV/AIDS	99					AIDS	Yes	AIDS	Yes
154	M	35.3	Pos	Pos	N19	B24	B23	Yes	HIV/AIDS	95					HIV/AIDS	99					Diarrhea/Dysentery	No	AIDS	Yes
155	F	30.2	Pos	Pos	A15	B20	B20	Yes	ARTI	86					Other cardiac dx	89					Undetermined	No	AIDS	Yes
156	M	29.5	Pos	Pos	A16	B20	B20	Yes	Pulmonary TB	100					Pulmonary TB	99					Diabetes	No	AIDS	Yes
157	M	44.6	Pos	Pos	A16	B20	B20	Yes	HIV/AIDS	60	Pulmonary TB	38			HIV/AIDS	99					Undetermined	No	AIDS	Yes
158	F	31.5	Pos	Pos	B24		B24	Yes	HIV/AIDS	100					HIV/AIDS	99					Diarrhea/Dysentery	No	AIDS	Yes
159	F	29.9	Pos	Pos	B24		B24	Yes	HIV/AIDS	100					HIV/AIDS	99					Undetermined	No	AIDS	Yes
160	F	41.6	Pos	Pos	A18	B20	B20	Yes	HIV/AIDS	100					HIV/AIDS	99					Pneumonia	No	AIDS	Yes

Participant details				VA	PCVA				InterVA-4						InterVA-5						SmartVA-Analyze v1.1.1		SmartVA-Analyze v.1.2.1	
ID	Sex	Age*	HIV status	HIV status	iCoD	uCoD	MMDS CoD	HIV CoD?	Cause 1	Lik 1	Cause 2	Lik 2	Cause 3	Lik 3	Cause 1	Lik 1	Cause 2	Lik 2	Cause 3	Lik 3	CoD	HIV CoD?	CoD	HIV CoD?
161	M	36.1	Pos	Pos	J15	B20	B20	Yes	Pulmonary TB	99					HIV/AIDS	99					Undetermined	No	AIDS	Yes
162	M	44.4	Pos	DK	J15	E11	J15	No	Other/unspecified infection	100					Other/unspecified infection	99					Diabetes	No	Diabetes	No
163	M	53.8	Pos	Pos	A15	B20	B20	Yes	HIV/AIDS	92					HIV/AIDS	92					Leukemia/Lymphomas	No	AIDS	Yes
164	M	48.3	Pos	Pos	A16	B20	B20	Yes	HIV/AIDS	83					HIV/AIDS	88					Undetermined	No	AIDS	Yes
165	F	49.7	Pos	DK	C50		C50	No	Breast Ca	85					ARTI	52	HIV/AIDS	26			Breast Cancer	No	Breast Cancer	No
166	F	38.8	Pos	Pos	A09	B20	A09	No	HIV/AIDS	96					HIV/AIDS	99					AIDS	Yes	AIDS	Yes
167	F	28.4	Pos	Pos	A16	B20	B20	Yes	Pulmonary TB	84					Pulmonary TB	43	HIV/AIDS	33			Undetermined	No	AIDS	Yes
168	F	41.9	Pos	Pos	A18	B20	B20	Yes	HIV/AIDS	100					HIV/AIDS	99					AIDS	Yes	AIDS	Yes
169	F	36.1	Pos	Pos	B24		B24	Yes	HIV/AIDS	72					HIV/AIDS	99					AIDS	Yes	AIDS	Yes
170	M	42.3	Pos	Pos	T96	A16	X44	No	Pulmonary TB	100					Pulmonary TB	98					Undetermined	No	AIDS	Yes
171	F	29.3	Pos	Pos	I26	B24	B23	Yes	Breast Ca	65	ARTI	34			ARTI	98					Undetermined	No	AIDS	Yes
172	M	34.1	Pos	Pos	A15	B20	B20	Yes	Pulmonary TB	100					Pulmonary TB	99					Asthma	No	AIDS	Yes
173	M	64.4	Pos	Pos	B24		B24	Yes	Pulmonary TB	86					HIV/AIDS	66	Pulmonary TB	33			AIDS	Yes	AIDS	Yes
174	F	40.9	Pos	Pos	A16	B20	B20	Yes	HIV/AIDS	80					Pulmonary TB	43	Stroke	41			Breast Cancer	No	AIDS	Yes
175	F	27.1	Pos	Pos	B59	B20	B20	Yes	ARTI	99					ARTI	90					Diarrhea/Dysentery	No	AIDS	Yes
176	M	42.4	Pos	Pos	A15	B20	B20	Yes	HIV/AIDS	91					HIV/AIDS	99					AIDS	Yes	AIDS	Yes
177	M	53.4	Pos	Pos	I64	A19	B20	Yes	HIV/AIDS	95					HIV/AIDS	67					Diabetes	No	AIDS	Yes
178	F	37.5	Pos	Pos	K71	B20	K71	No	Diarrhoeal dx	80					Diarrhoeal dx	96					Diarrhea/Dysentery	No	AIDS	Yes
179	F	45.9	Pos	Pos	A16	B20	B20	Yes	HIV/AIDS	100					HIV/AIDS	99					Breast Cancer	No	AIDS	Yes
180	F	40.4	Pos	Pos	A16	B20	B20	Yes	HIV/AIDS	60	Pulmonary TB	38			HIV/AIDS	96					Undetermined	No	AIDS	Yes
181	M	48.7	Pos	Pos	B20		B20	Yes	HIV/AIDS	100					HIV/AIDS	99					AIDS	Yes	AIDS	Yes
182	F	53.9	Pos	Pos	A15	B20	B20	Yes	HIV/AIDS	100					HIV/AIDS	96					Diabetes	No	AIDS	Yes
183	M	63.1	Pos	Pos	B24		B24	Yes	HIV/AIDS	85					Digestive Ca	52					TB	No	AIDS	Yes

Participant details				VA	PCVA				InterVA-4						InterVA-5						SmartVA-Analyze v1.1.1		SmartVA-Analyze v.1.2.1	
ID	Sex	Age*	HIV status	HIV status	iCoD	uCoD	MMDS CoD	HIV CoD?	Cause 1	Lik 1	Cause 2	Lik 2	Cause 3	Lik 3	Cause 1	Lik 1	Cause 2	Lik 2	Cause 3	Lik 3	CoD	HIV CoD?	CoD	HIV CoD?
184	F	45.4	Pos	Pos	A09	B20	A09	No	HIV/AIDS	100					HIV/AIDS	99					Undetermined	No	AIDS	Yes
185	F	43.0	Pos	Pos	B20		B20	Yes	HIV/AIDS	100					HIV/AIDS	99					Undetermined	No	AIDS	Yes
186	F	36.5	Pos	Pos	B20		B20	Yes	HIV/AIDS	100					HIV/AIDS	99					AIDS	Yes	AIDS	Yes
187	F	43.1	Pos	DK	K25		K25	No	HIV/AIDS	99					HIV/AIDS	82					Breast Cancer	No	Undetermined	No
188	M	57.0	Pos	Pos	V03		X59	No	RTA	86					RTA	85					Road Traffic	No	Other Non-communicable Diseases	No
189	M	50.9	Pos	Neg	A16		A16	No	Pulmonary TB	100					Pulmonary TB	96					COPD	No	TB	No
190	M	33.8	Pos	Pos	B49	B20	B20	Yes	HIV/AIDS	56	Digestive Ca	44			HIV/AIDS	88					Undetermined	No	AIDS	Yes
191	F	26.7	Pos	Pos	N18	B24	B23	Yes	HIV/AIDS	96					HIV/AIDS	99					Diarrhea/Dysentery	No	AIDS	Yes
192	M	51.6	Pos	Neg	I50		I50	No	ARTI	56	HIV/AIDS	44			HIV/AIDS	99					Diabetes	No	Diabetes	No
193	M	36.7	Pos	Pos	K92	A15	B20	Yes	HIV/AIDS	100					HIV/AIDS	99					Leukemia/Lymphomas	No	AIDS	Yes
194	M	41.7	Pos	Pos	A16	B20	B20	Yes	Digestive Ca	95					Digestive Ca	52	HIV/AIDS	42			Colorectal Cancer	No	AIDS	Yes
195	F	57.6	Pos	Neg	E11		E11	No	HIV/AIDS	100					HIV/AIDS	83					Undetermined	No	Diabetes	No
196	M	51.6	Pos	Pos	A16	B20	B20	Yes	Pulmonary TB	97					Pulmonary TB	49	Digestive Ca	48			Undetermined	No	AIDS	Yes
197	F	32.0	Pos	Neg	K27	A18	B20	Yes	Digestive Ca	99					Digestive Ca	95					Other Non-communicable Diseases	No	TB	No
198	F	38.8	Pos	Pos	A16	B20	B20	Yes	HIV/AIDS	99					HIV/AIDS	98					AIDS	Yes	AIDS	Yes
199	F	31.3	Pos	Neg	K56		K56	No	Acute abdomen	100					Acute abdomen	99					Maternal	No	TB	No
200	F	40.1	Pos	Pos	A16	B20	B20	Yes	HIV/AIDS	100					HIV/AIDS	99					AIDS	Yes	Breast Cancer	No
201	M	45.8	Pos	Pos	A15	B20	B20	Yes	Digestive Ca	100					Digestive Ca	99					Other Non-communicable Diseases	No	AIDS	Yes
202	F	39.4	Pos	Pos	A16	B20	B20	Yes	HIV/AIDS	99					HIV/AIDS	99					Undetermined	No	AIDS	Yes
203	M	27.4	Pos	Pos	J18	B20	B20	Yes	HIV/AIDS	100					HIV/AIDS	99					Undetermined	No	AIDS	Yes

Participant details				VA HIV status	PCVA				InterVA-4						InterVA-5						SmartVA-Analyze v1.1.1		SmartVA-Analyze v.1.2.1	
ID	Sex	Age*	HIV status		iCoD	uCoD	MMDS CoD	HIV CoD?	Cause 1	Lik 1	Cause 2	Lik 2	Cause 3	Lik 3	Cause 1	Lik 1	Cause 2	Lik 2	Cause 3	Lik 3	CoD	HIV CoD?	CoD	HIV CoD?
204	F	26.4	Pos	Pos	A18	B20	B20	Yes	Pulmonary TB	95					Pulmonary TB	99					Breast Cancer	No	AIDS	Yes
205	F	64.1	Pos	Pos	A09	B20	A09	No	HIV/AIDS	100					HIV/AIDS	99					Leukemia/Lymphomas	No	AIDS	Yes
206	F	33.6	Pos	Neg	B24		B24	Yes	HIV/AIDS	99					HIV/AIDS	98					AIDS	Yes	Undetermined	No
207	M	49.7	Pos	Pos	J15	B20	B20	Yes	HIV/AIDS	98					HIV/AIDS	99					Undetermined	No	AIDS	Yes
208	M	37.8	Pos	Pos	A16	B20	B20	Yes	Pulmonary TB	91					HIV/AIDS	93					Asthma	No	AIDS	Yes
209	F	50.7	Pos	DK	A19	B20	B20	Yes	HIV/AIDS	100					HIV/AIDS	100					Leukemia/Lymphomas	No	Leukemia/Lymphomas	No
210	F	35.2	Pos	Pos	O88	B24	B23	Yes	Cirrhosis	65					Stroke	50	Cirrhosis	40			Undetermined	No	AIDS	Yes
211	F	43.4	Pos	Pos	A16	B20	B20	Yes	Pulmonary TB	96					HIV/AIDS	92					Breast Cancer	No	AIDS	Yes
212	M	34.2	Pos	Pos	N17	A16	B20	Yes	ARTI	100					ARTI	96					AIDS	Yes	AIDS	Yes
213	M	30.2	Pos	Neg	A16	NA	A16	No	Pulmonary TB	100					Pulmonary TB	99					TB	No	TB	No
214	M	34.1	Pos	Pos	G03	B20	B20	Yes	Stroke	57					Self-harm	47	Meningitis/encephalitis	23			Undetermined	No	AIDS	Yes
215	M	57.6	Pos	DK	K70	NA	K70	No	Digestive Ca	100					Digestive Ca	99					Other Non-communicable Diseases	No	Other Non-communicable Diseases	No
216	F	52.6	Pos	Pos	B24	NA	B24	Yes	Pulmonary TB	93					HIV/AIDS	51	Pulmonary TB	41			Undetermined	No	AIDS	Yes
217	M	70.0	Pos	Neg	K76	C18	C18	No	Digestive Ca	100					Digestive Ca	99					Other Non-communicable Diseases	No	Other Non-communicable Diseases	No
218	M	39.0	Pos	Pos	A15	B20	B20	Yes	Other/unspecified infection	95					Other/unspecified infection	98					AIDS	Yes	AIDS	Yes
219	F	54.5	Pos	Pos	J18	B20	B20	Yes	HIV/AIDS	100					HIV/AIDS	99					Undetermined	No	AIDS	Yes
220	F	53.0	Neg	Neg	A16	NA	A16	No	Pulmonary TB	100					Pulmonary TB	99					IHD - Acute Myocardial Infarction	No	TB	No
221	F	44.6	Pos	Pos	A18	B20	B20	Yes	HIV/AIDS	100					HIV/AIDS	99					Leukemia/Lymphomas	No	AIDS	Yes
222	F	35.5	Pos	Pos	A16	B20	B20	Yes	HIV/AIDS	100					HIV/AIDS	99					AIDS	Yes	AIDS	Yes
223	F	59.6	Pos	Pos	J18	NA	J18	No	HIV/AIDS	100					HIV/AIDS	99					Undetermined	No	AIDS	Yes

Participant details				VA	PCVA				InterVA-4						InterVA-5						SmartVA-Analyze v1.1.1		SmartVA-Analyze v.1.2.1	
ID	Sex	Age*	HIV status	HIV status	iCoD	uCoD	MMDS CoD	HIV CoD?	Cause 1	Lik 1	Cause 2	Lik 2	Cause 3	Lik 3	Cause 1	Lik 1	Cause 2	Lik 2	Cause 3	Lik 3	CoD	HIV CoD?	CoD	HIV CoD?
224	M	35.2	Pos	Pos	X70	NA	W76	No	Self-harm	100					Self-harm	99					Suicide	No	Road Traffic	No
225	F	31.3	Pos	Pos	A19	B20	B20	Yes	HIV/AIDS	92					HIV/AIDS	99					AIDS	Yes	AIDS	Yes
226	M	36.0	Pos	DK	A17	B20	B20	Yes	Haemorrhagic fever	56	HIV/AIDS	44			HIV/AIDS	96					Leukemia/Lymphomas	No	Other Non-communicable Diseases	No
227	F	22.7	Neg	Pos	R99	B20	B20	Yes	Indeterminate	100					HIV/AIDS	94					Epilepsy	No	AIDS	Yes
228	M	50.8	Pos	Pos	R04	A16	B20	Yes	Pulmonary TB	67					HIV/AIDS	99					Lung Cancer	No	AIDS	Yes
229	F	25.6	Pos	Pos	D64	B24	B23	Yes	HIV/AIDS	100					HIV/AIDS	99					Diarrhea/Dysentery	No	AIDS	Yes
230	F	51.0	Pos	DK	R19	B24	B24	Yes	Other Ca	59	HIV/AIDS	38			Reproductive Ca	45	HIV/AIDS	36			Undetermined	No	Undetermined	No
231	F	34.8	Pos	Pos	A16	B20	B20	Yes	Pulmonary TB	86					HIV/AIDS	86					Undetermined	No	AIDS	Yes
232	F	48.8	Pos	Pos	J18	A06	A09	No	Acute abdomen	58					Cirrhosis	88					AIDS	Yes	Stroke	No
233	F	50.4	Pos	Pos	B59	B20	B20	Yes	HIV/AIDS	100					HIV/AIDS	99					Cervical Cancer	No	AIDS	Yes
234	M	24.2	Pos	Pos	A16	B20	B20	Yes	HIV/AIDS	100					HIV/AIDS	99					Other Infectious Diseases	No	AIDS	Yes
235	M	33.3	Pos	Pos	A16	B20	B20	Yes	Pulmonary TB	100					Pulmonary TB	99					Leukemia/Lymphomas	No	AIDS	Yes
236	F	25.9	Pos	Pos	I64	A15	B23	Yes	HIV/AIDS	100					HIV/AIDS	99					AIDS	Yes	AIDS	Yes
237	F	28.1	Pos	Pos	A19	B20	B20	Yes	HIV/AIDS	92					HIV/AIDS	99					Leukemia/Lymphomas	No	AIDS	Yes
238	M	27.5	Pos	Pos	A16	B20	B20	Yes	Pulmonary TB	100					Pulmonary TB	98					TB	No	AIDS	Yes
239	M	41.5	Pos	Pos	A17	B20	B20	Yes	Pulmonary TB	94					Pulmonary TB	71					Leukemia/Lymphomas	No	AIDS	Yes
240	F	51.4	Pos	Pos	A16	NA	A16	No	Pulmonary TB	100					Pulmonary TB	99					Undetermined	No	AIDS	Yes
241	M	31.8	Pos	DK	A17	B20	B20	Yes	HIV/AIDS	83					HIV/AIDS	99					AIDS	Yes	AIDS	Yes
242	M	42.6	Pos	Pos	A19	B20	B20	Yes	Pulmonary TB	99					HIV/AIDS	99					Undetermined	No	AIDS	Yes
243	F	39.7	Pos	Pos	A17	B20	B20	Yes	HIV/AIDS	62					Stroke	55	HIV/AIDS	44			Breast Cancer	No	AIDS	Yes
244	F	57.9	Pos	Neg	I64	NA	I64	No	HIV/AIDS	100					HIV/AIDS	99					Epilepsy	No	AIDS	Yes
245	M	37.3	Pos	Pos	B24	NA	B24	Yes	Pulmonary TB	96					HIV/AIDS	81					Undetermined	No	AIDS	Yes

Participant details				VA	PCVA				InterVA-4						InterVA-5						SmartVA-Analyze v1.1.1		SmartVA-Analyze v.1.2.1	
ID	Sex	Age*	HIV status	HIV status	iCoD	uCoD	MMDS CoD	HIV CoD?	Cause 1	Lik 1	Cause 2	Lik 2	Cause 3	Lik 3	Cause 1	Lik 1	Cause 2	Lik 2	Cause 3	Lik 3	CoD	HIV CoD?	CoD	HIV CoD?
246	M	29.7	Pos	Pos	A17	B20	B20	Yes	Oral Ca	100					Oral Ca	96					Leukemia/Lymphomas	No	AIDS	Yes
247	M	65.8	Neg	Pos	C16	NA	C16	No	Other Ca	70					Digestive Ca	50	Other Ca	49			Diarrhea/Dysentery	No	AIDS	Yes
248	M	28.5	Pos	Pos	A16	B20	B20	Yes	Pulmonary TB	45	HIV/AIDS	23	Other Ca	23	HIV/AIDS	99					Diabetes	No	AIDS	Yes
249	F	32.1	Pos	Pos	A17	B20	B20	Yes	Pulmonary TB	100					Pulmonary TB	60	HIV/AIDS	37			AIDS	Yes	AIDS	Yes
250	M	61.9	Neg	Pos	A16	B24	B20	Yes	HIV/AIDS	100					HIV/AIDS	99					Diarrhea/Dysentery	No	AIDS	Yes
251	F	43.4	Pos	Pos	D68	NA	D68	No	HIV/AIDS	100					HIV/AIDS	99					AIDS	Yes	AIDS	Yes
252	F	40.1	Pos	Pos	K63	N17	B23	Yes	Digestive Ca	76					Cirrhosis	54	HIV/AIDS	44			Diarrhea/Dysentery	No	AIDS	Yes
253	F	77.3	Neg	Pos	I50	NA	I50	No	Pulmonary TB	100					Pulmonary TB	97					Undetermined	No	TB	No
254	F	38.8	Pos	Pos	G00	B20	B20	Yes	HIV/AIDS	96					HIV/AIDS	99					AIDS	Yes	AIDS	Yes
255	F	39.3	Pos	Pos	A18	B20	B20	Yes	Pulmonary TB	97					HIV/AIDS	82					Breast Cancer	No	AIDS	Yes
256	M	33.4	Neg	Pos	A16	B20	B20	Yes	Pulmonary TB	72					HIV/AIDS	99					AIDS	Yes	AIDS	Yes
257	M	39.7	Pos	Pos	K29	B20	B20	Yes	HIV/AIDS	41	Oral Ca	32	Other Ca	25	HIV/AIDS	99					AIDS	Yes	AIDS	Yes
258	M	30.0	Pos	Pos	A17	B20	B20	Yes	Digestive Ca	60	Pulmonary TB	39			Digestive Ca	96					TB	No	AIDS	Yes
259	F	57.5	Pos	Pos	C53	NA	C53	No	HIV/AIDS	76					Reproductive Ca	97					IHD - Acute Myocardial Infarction	No	AIDS	Yes
260	M	43.2	Pos	Pos	B24	NA	B24	Yes	Pulmonary TB	84					HIV/AIDS	70					Diabetes	No	AIDS	Yes
261	M	33.9	Pos	Pos	B24	NA	B24	Yes	Pulmonary TB	100					Pulmonary TB	99					Undetermined	No	AIDS	Yes
262	F	30.7	Pos	Pos	A15	B20	B20	Yes	Digestive Ca	100					Digestive Ca	99					Diarrhea/Dysentery	No	AIDS	Yes
263	F	50.6	Pos	Pos	B24	NA	B24	Yes	HIV/AIDS	99					HIV/AIDS	99					Breast Cancer	No	AIDS	Yes
264	M	46.8	Pos	Pos	I64	B23	B23	Yes	Acute cardiac dx	86					Acute cardiac dx	97					Undetermined	No	AIDS	Yes
265	F	42.2	Pos	Pos	B21	NA	B21	Yes	Pulmonary TB	96					Pulmonary TB	94					Undetermined	No	AIDS	Yes

Participant details				VA	PCVA				InterVA-4						InterVA-5						SmartVA-Analyze v1.1.1		SmartVA-Analyze v.1.2.1	
ID	Sex	Age*	HIV status	HIV status	iCoD	uCoD	MMDS CoD	HIV CoD?	Cause 1	Lik 1	Cause 2	Lik 2	Cause 3	Lik 3	Cause 1	Lik 1	Cause 2	Lik 2	Cause 3	Lik 3	CoD	HIV CoD?	CoD	HIV CoD?
266	F	44.4	Pos	DK	A16	B20	B20	Yes	Pulmonary TB	44	COPD	34			Other cardiac dx	98					AIDS	Yes	Undetermined	No
267	M	36.0	Pos	DK	J18	B20	B20	Yes	Respiratory Ca	55	Pulmonary TB	45			Respiratory Ca	75					AIDS	Yes	Undetermined	No
268	M	56.0	Pos	Pos	C80	NA	C80	No	COPD	100					COPD	99					COPD	No	AIDS	Yes
269	M	36.6	Pos	Pos	D68	B23	B23	Yes	HIV/AIDS	100					HIV/AIDS	83					AIDS	Yes	AIDS	Yes
270	F	39.4	Pos	Pos	B24	NA	B24	Yes	HIV/AIDS	73					Stroke	99					Breast Cancer	No	AIDS	Yes
271	M	36.6	Pos	Pos	G03	B20	B20	Yes	Acute abdomen	50	Cirrhosis	31			HIV/AIDS	96					Undetermined	No	AIDS	Yes
272	M	29.6	Pos	Pos	A16	B20	B20	Yes	Pulmonary TB	100					Pulmonary TB	99					Lung Cancer	No	AIDS	Yes
273	M	51.1	Pos	Pos	A09	B20	A09	No	Digestive Ca	51	Cirrhosis	33			Digestive Ca	90					COPD	No	AIDS	Yes
274	F	53.8	Pos	DK	A17	B20	B20	Yes	HIV/AIDS	94					HIV/AIDS	96					Undetermined	No	Undetermined	No
275	F	26.9	Pos	Neg	K92	NA	K92	No	Pulmonary TB	95					Pulmonary TB	55	ARTI	43			Undetermined	No	Undetermined	No
276	M	40.0	Pos	Pos	A16	B20	B20	Yes	Pulmonary TB	62	ARTI	38			Pulmonary TB	92					AIDS	Yes	AIDS	Yes
277	F	34.0	Pos	DK	B24	NA	B24	Yes	HIV/AIDS	99					HIV/AIDS	99					Undetermined	No	Undetermined	No
278	M	56.2	Neg	Neg	A16	NA	A16	No	Acute cardiac dx	95					Acute cardiac dx	94					Undetermined	No	Undetermined	No
279	M	34.9	Pos	Pos	A16	B20	B20	Yes	Pulmonary TB	100					Pulmonary TB	99					Undetermined	No	AIDS	Yes
280	F	29.4	Pos	Pos	B24	NA	B24	Yes	HIV/AIDS	100					HIV/AIDS	99					Diarrhea/Dysentery	No	AIDS	Yes
281	M	45.0	Pos	Pos	G00	B20	B20	Yes	Diabetes	79					Diabetes	99					Epilepsy	No	AIDS	Yes
282	M	41.5	Pos	Pos	G00	B20	B20	Yes	Other Ca	44	Other NCD	28			Other Ca	94					AIDS	Yes	AIDS	Yes
283	F	40.5	Pos	DK	A16	B20	B20	Yes	HIV/AIDS	67					HIV/AIDS	71					AIDS	Yes	AIDS	Yes
284	M	42.3	Pos	Neg	A16	B20	B20	Yes	Pulmonary TB	100					Pulmonary TB	99					AIDS	Yes	TB	No
285	M	37.6	Pos	Pos	G00	B20	B20	Yes	HIV/AIDS	95					HIV/AIDS	86					TB	No	AIDS	Yes
286	M	51.4	Pos	Pos	A15	B20	B20	Yes	Digestive Ca	98					HIV/AIDS	62	Digestive Ca	37			Lung Cancer	No	AIDS	Yes
287	M	46.5	Pos	Pos	A09	B20	A09	No	HIV/AIDS	100					HIV/AIDS	99					AIDS	Yes	AIDS	Yes

Participant details				VA	PCVA				InterVA-4						InterVA-5						SmartVA-Analyze v1.1.1		SmartVA-Analyze v.1.2.1	
ID	Sex	Age*	HIV status	HIV status	iCoD	uCoD	MMDS CoD	HIV CoD ?	Cause 1	Lik 1	Cause 2	Lik 2	Cause 3	Lik 3	Cause 1	Lik 1	Cause 2	Lik 2	Cause 3	Lik 3	CoD	HIV CoD ?	CoD	HIV CoD ?
288	F	29.0	Pos	Pos	A18	B20	B20	Yes	Pulmonary TB	93					HIV/AIDS	85					AIDS	Yes	AIDS	Yes
289	F	34.3	Pos	DK	A16	B20	B20	Yes	Digestive Ca	79					HIV/AIDS	99					AIDS	Yes	AIDS	Yes
290	F	30.1	Pos	Pos	A15	B20	B20	Yes	HIV/AIDS	100					HIV/AIDS	99					Breast Cancer	No	AIDS	Yes
291	F	37.2	Pos	Neg	D64		D64	No	Diarrhoeal dx	100					Diarrhoeal dx	99					Undetermined	No	Diarrhea/Dysentery	No
292	F	32.9	Pos	DK	B24		B24	Yes	HIV/AIDS	100					HIV/AIDS	99					Diarrhea/Dysentery	No	Diabetes	No
293	F	44.2	Pos	Pos	A16	B20	B20	Yes	Acute cardiac dx	60					Acute cardiac dx	96					Undetermined	No	AIDS	Yes
294	M	41.7	Pos	Pos	A16	B20	B20	Yes	Pulmonary TB	100					Pulmonary TB	50	HIV/AIDS	49			AIDS	Yes	AIDS	Yes
295	M	31.8	Pos	DK	A16	B20	B20	Yes	Pulmonary TB	100					Pulmonary TB	99					Undetermined	No	TB	No
296	M	52.3	Pos	DK	A16	NA	A16	No	Pulmonary TB	100					Pulmonary TB	99					Asthma	No	TB	No
297	M	41.3	Pos	Pos	B24	NA	B24	Yes	HIV/AIDS	100					HIV/AIDS	98					AIDS	Yes	AIDS	Yes
298	F	24.6	Pos	Pos	K56	NA	K56	No	HIV/AIDS	100					HIV/AIDS	99					Leukemia/Lymphomas	No	AIDS	Yes
299	F	57.8	Pos	Pos	A16	B20	B20	Yes	Pulmonary TB	75					Diabetes	92					Undetermined	No	AIDS	Yes
300	M	67.0	Pos	Pos	A16	B20	B20	Yes	Pulmonary TB	99					Other Ca	54	Pulmonary TB	45			COPD	No	AIDS	Yes
301	F	31.4	Pos	Pos	E86	B20	B23	Yes	HIV/AIDS	100					HIV/AIDS	98					Undetermined	No	AIDS	Yes
302	M	32.6	Pos	Pos	B24	NA	B24	Yes	HIV/AIDS	100					HIV/AIDS	95					Undetermined	No	AIDS	Yes
303	M	29.3	Pos	Pos	S06	NA	G93	No	HIV/AIDS	97					Pulmonary TB	75					Undetermined	No	AIDS	Yes
304	M	28.9	Pos	Pos	A19	B20	B20	Yes	Indeterminate						Indeterminate	0					Undetermined	No	AIDS	Yes
305	F	37.9	Pos	Pos	A15	B20	B20	Yes	Pulmonary TB	100					Pulmonary TB	83					Asthma	No	AIDS	Yes
306	M	47.5	Pos	Neg	A15	NA	A16	No	Pulmonary TB	86					HIV/AIDS	99					Leukemia/Lymphomas	No	TB	No
307	F	40.7	Pos	Pos	A16	B20	B20	Yes	HIV/AIDS	56	Pulmonary TB	44			HIV/AIDS	99					AIDS	Yes	AIDS	Yes
308	M	42.6	Pos	Pos	A18	B20	B20	Yes	ARTI	100					ARTI	99					Asthma	No	AIDS	Yes

Participant details				VA	PCVA				InterVA-4						InterVA-5						SmartVA-Analyze v1.1.1		SmartVA-Analyze v.1.2.1	
ID	Sex	Age*	HIV status	HIV status	iCoD	uCoD	MMDS CoD	HIV CoD?	Cause 1	Lik 1	Cause 2	Lik 2	Cause 3	Lik 3	Cause 1	Lik 1	Cause 2	Lik 2	Cause 3	Lik 3	CoD	HIV CoD?	CoD	HIV CoD?
309	F	40.1	Pos	Pos	A19	B20	B20	Yes	Acute abdomen	93					HIV/AIDS	99					Undetermined	No	AIDS	Yes
310	M	69.7	Neg	Pos	T65	A18	X49	No	Pulmonary TB	100					Pulmonary TB	99					Asthma	No	AIDS	Yes
311	F	44.5	Pos	Neg	B24	NA	B24	Yes	HIV/AIDS	100					HIV/AIDS	99					Breast Cancer	No	Undetermined	No
312	F	43.4	Pos	Pos	A17	B20	B20	Yes	Pulmonary TB	100					Pulmonary TB	99					Pneumonia	No	AIDS	Yes
313	F	54.2	Pos	Pos	B24	NA	B24	Yes	Diarrhoeal dx	61	HIV/AIDS	39			Diarrhoeal dx	60	HIV/AIDS	39			Epilepsy	No	AIDS	Yes
314	M	42.3	Pos	Pos	A18	B20	B20	Yes	HIV/AIDS	44	Digestive Ca	43			HIV/AIDS	75					AIDS	Yes	AIDS	Yes
315	M	25.8	Pos	Pos	A19	B20	B20	Yes	HIV/AIDS	100					HIV/AIDS	99					Pneumonia	No	AIDS	Yes
316	F	40.0	Pos	Pos	A16	B20	B20	Yes	ARTI	100					HIV/AIDS	61	ARTI	38			Breast Cancer	No	AIDS	Yes
317	F	42.5	Pos	Pos	B24	NA	B24	Yes	Acute abdomen	100					Acute abdomen	99					Undetermined	No	AIDS	Yes
318	F	26.7	Pos	Pos	J18	A09	A09	No	HIV/AIDS	100					HIV/AIDS	97					Stroke	No	AIDS	Yes
319	M	73.1	Pos	Pos	A16	B20	B20	Yes	Severe malnutrition	83					HIV/AIDS	99					Asthma	No	AIDS	Yes
320	F	23.5	Pos	DK	B24	NA	B24	Yes	HIV/AIDS	56	Pulmonary TB	44			HIV/AIDS	54	Pulmonary TB	43			Undetermined	No	Chronic Respiratory	No
321	F	60.0	Pos	DK	K29	K70	K29	No	Other cardiac dx	99					Other cardiac dx	99					Lung Cancer	No	Ischemic Heart Disease	No
322	M	52.3	Pos	Pos	X49	NA	X49	No	Indeterminate	100					Acute cardiac dx	99					Undetermined	No	AIDS	Yes
323	F	50.7	Pos	Pos	A15	B20	B20	Yes	HIV/AIDS	99					HIV/AIDS	99					Breast Cancer	No	AIDS	Yes
324	M	41.6	Pos	Pos	I50	B24	B23	Yes	Other cardiac dx	94					Other cardiac dx	88					Leukemia/Lymphomas	No	AIDS	Yes
325	M	49.8	Pos	Pos	N18	B24	B23	Yes	Digestive Ca	100					Digestive Ca	96					Leukemia/Lymphomas	No	AIDS	Yes
326	F	39.2	Pos	Neg	N18		N18	No	HIV/AIDS	100					HIV/AIDS	99					Breast Cancer	No	Undetermined	No
327	M	54.2	Pos	DK	I26	S72	X59	No	Accidental fall	76					Accidental fall	75					Falls	No	Undetermined	No
328	M	47.4	Pos	Pos	N19	B24	B23	Yes	Renal failure	100					Renal failure	99					Undetermined	No	AIDS	Yes
329	M	62.8	Pos	Neg	N18	N36	N35	No	ARTI	100					ARTI	96					Undetermined	No	Undetermined	No

Participant details				VA	PCVA				InterVA-4						InterVA-5						SmartVA-Analyze v1.1.1		SmartVA-Analyze v.1.2.1	
ID	Sex	Age*	HIV status	HIV status	iCoD	uCoD	MMDS CoD	HIV CoD?	Cause 1	Lik 1	Cause 2	Lik 2	Cause 3	Lik 3	Cause 1	Lik 1	Cause 2	Lik 2	Cause 3	Lik 3	CoD	HIV CoD?	CoD	HIV CoD?
330	F	47.3	Pos	Pos	I64	B24	B23	Yes	HIV/AIDS	100					HIV/AIDS	99					Breast Cancer	No	Breast Cancer	No
331	F	44.5	Pos	Pos	N18	B24	B23	Yes	HIV/AIDS	95					HIV/AIDS	85					Diabetes	No	AIDS	Yes
332	F	45.2	Pos	Pos	N19	B24	B23	Yes	HIV/AIDS	100					HIV/AIDS	99					Diabetes	No	AIDS	Yes
333	F	39.0	Pos	DK	C79	C50	C50	No	Reproductive Ca	96					Digestive Ca	41	Stroke	32	Reproductive Ca	26	Undetermined	No	Cervical Cancer	No
334	M	34.6	Pos	Pos	C96	B21	C49	No	HIV/AIDS	76					Other Ca	85					Diarrhea/Dysentery	No	AIDS	Yes
335	F	30.4	Pos	Pos	J22	B20	B20	Yes	ARTI	100					HIV/AIDS	56	ARTI	43			Undetermined	No	AIDS	Yes
336	M	64.9	Pos	Neg	L03	E11	L03	No	Other Ca	90					Other Ca	69					Undetermined	No	Esophageal Cancer	No
337	M	41.2	Pos	Pos	N19	B24	B23	Yes	Other Ca	89					HIV/AIDS	99					AIDS	Yes	AIDS	Yes
338	F	38.9	Pos	Pos	C69	B21	C69	No	Oral Ca	99					Oral Ca	81					Undetermined	No	AIDS	Yes
339	F	43.3	Pos	Pos	S09		X59	No	HIV/AIDS	100					HIV/AIDS	99					Breast Cancer	No	AIDS	Yes
340	M	40.3	Pos	Pos	N18		N18	No	HIV/AIDS	89					Reproductive Ca	90					Undetermined	No	AIDS	Yes
341	M	39.3	Pos	Pos	A09	B20	A09	No	Digestive Ca	100					Digestive Ca	99					AIDS	Yes	AIDS	Yes
342	F	57.7	Pos	DK	C57		C80	No	Respiratory Ca	99					Respiratory Ca	99					Asthma	No	Lung Cancer	No
343	F	34.7	Pos	Pos	J18	B20	B20	Yes	HIV/AIDS	68					Acute cardiac dx	53					Undetermined	No	AIDS	Yes
344	F	53.2	Pos	Pos	I50	B24	B23	Yes	Reproductive Ca	99					Reproductive Ca	96					Cervical Cancer	No	Cervical Cancer	No
345	F	52.1	Pos	Pos	I64	I10	I64	No	Pulmonary TB	100					Pulmonary TB	98					IHD - Acute Myocardial Infarction	No	AIDS	Yes
346	F	28.5	Pos	Neg	D68	N18	D68	No	HIV/AIDS	100					HIV/AIDS	99					Stomach Cancer	No	Undetermined	No
347	F	35.0	Pos	Pos	C92	B24	B21	Yes	Digestive Ca	100					Digestive Ca	99					Diarrhea/Dysentery	No	AIDS	Yes
348	F	40.9	Pos	DK	B24		B24	Yes	Digestive Ca	100					Digestive Ca	99					Undetermined	No	Undetermined	No
349	F	45.7	Pos	Pos	C83	B21	B21	Yes	Reproductive Ca	99					Reproductive Ca	99					Breast Cancer	No	Breast Cancer	No
350	M	37.5	Pos	Pos	A16	B20	B20	Yes	Pulmonary TB	51	ARTI	49			ARTI	97					Undetermined	No	AIDS	Yes
351	M	58.0	Pos	Pos	A16	B20	B20	Yes	Digestive Ca	100					Digestive Ca	99					Diarrhea/Dysentery	No	AIDS	Yes
352	F	59.6	Pos	Pos	A16	B20	B20	Yes	ARTI	100					Diabetes	92					Diabetes	No	AIDS	Yes

Participant details				VA	PCVA				InterVA-4						InterVA-5						SmartVA-Analyze v1.1.1		SmartVA-Analyze v.1.2.1	
ID	Sex	Age*	HIV status	HIV status	iCoD	uCoD	MMDS CoD	HIV CoD?	Cause 1	Lik 1	Cause 2	Lik 2	Cause 3	Lik 3	Cause 1	Lik 1	Cause 2	Lik 2	Cause 3	Lik 3	CoD	HIV CoD?	CoD	HIV CoD?
353	F	57.8	Pos	Pos	K92	B20	B23	Yes	HIV/AIDS	98					HIV/AIDS	99					Diabetes	No	AIDS	Yes
354	F	47.2	Pos	Pos	D68	B24	B23	Yes	HIV/AIDS	97					HIV/AIDS	70					AIDS	Yes	AIDS	Yes
355	F	44.6	Pos	Pos	A09	B20	A09	No	ARTI	100					ARTI	99					AIDS	Yes	AIDS	Yes
356	F	29.5	Pos	Pos	A09	B20	A09	No	HIV/AIDS	89					HIV/AIDS	95					Diabetes	No	Diabetes	No
357	F	65.8	Pos	Pos	A09	B20	A09	No	HIV/AIDS	99					HIV/AIDS	91					Undetermined	No	AIDS	Yes
358	F	23.9	Pos	Pos	A16	B20	B20	Yes	Asthma	75					Other cardiac dx	84					Undetermined	No	AIDS	Yes
359	F	38.8	Pos	Pos	E87	B24	B23	Yes	HIV/AIDS	100					HIV/AIDS	99					Diarrhea/Dysentery	No	AIDS	Yes
360	F	48.2	Pos	Pos	A17	B20	B20	Yes	HIV/AIDS	100					HIV/AIDS	99					Undetermined	No	AIDS	Yes
361	F	33.5	Pos	Pos	A16	B20	B20	Yes	Pulmonary TB	56	HIV/AIDS	44			HIV/AIDS	99					Undetermined	No	AIDS	Yes
362	M	37.2	Pos	Pos	A15	B20	B20	Yes	Pulmonary TB	100					Pulmonary TB	98					Diabetes	No	AIDS	Yes
363	M	51.4	Pos	Pos	A15	B20	B20	Yes	ARTI	100					ARTI	99					TB	No	AIDS	Yes
364	F	54.0	Pos	Pos	B24	E11	B24	Yes	Diabetes	86					Diabetes	99					Diabetes	No	AIDS	Yes
365	M	51.5	Neg	DK	A19		A19	No	Stroke	98					Stroke	67					Lung Cancer	No	Undetermined	No
366	F	58.9	Neg	Neg	N18	E11	E11	No	Other cardiac dx	58	Diabetes	37			Other cardiac dx	60	Diabetes	38			Undetermined	No	Diabetes	No
367	M	49.5	Neg	Neg	K70		K70	No	Digestive Ca	100					Digestive Ca	99					AIDS	Yes	Undetermined	No
368	M	67.0	Neg	Neg	A16	E11	A16	No	Acute abdomen	69					Acute cardiac dx	64					Lung Cancer	No	TB	No
369	M	66.6	Neg	Neg	I50	I10	I50	No	Digestive Ca	100					Digestive Ca	99					COPD	No	Ischemic Heart Disease	No
370	F	50.9	Neg	Neg	I50	E11	I50	No	Diabetes	98					Diabetes	98					Undetermined	No	Diabetes	No
371	F	55.9	Neg	Neg	L53	A16	L53	No	HIV/AIDS	100					HIV/AIDS	99					Diabetes	No	TB	No
372	M	36.0	Neg	Neg	I50		I50	No	HIV/AIDS	84					HIV/AIDS	98					Diabetes	No	Diabetes	No
373	M	47.2	Neg	Neg	C44		C80	No	Oral Ca	53	Other Ca	42			Other Ca	84					Leukemia/Lymphomas	No	Leukemia/Lymphomas	No
374	M	19.6	Neg	Neg	N19		N19	No	Other cardiac dx	56	Renal failure	44			Renal failure	88					Renal Failure	No	Ischemic Heart Disease	No
375	M	51.9	Neg	Neg	G95		G95	No	Acute abdomen	100					Acute abdomen	99					Undetermined	No	Undetermined	No

Participant details				VA	PCVA				InterVA-4						InterVA-5						SmartVA-Analyze v1.1.1		SmartVA-Analyze v.1.2.1	
ID	Sex	Age*	HIV status	HIV status	iCoD	uCoD	MMDS CoD	HIV CoD ?	Cause 1	Lik 1	Cause 2	Lik 2	Cause 3	Lik 3	Cause 1	Lik 1	Cause 2	Lik 2	Cause 3	Lik 3	CoD	HIV CoD ?	CoD	HIV CoD ?
376	F	34.0	Neg	Neg	O90		O95	No	ARTI	100					ARTI	99					Undetermined	No	Undetermined	No
377	M	57.4	Neg	Neg	I50		I50	No	Other cardiac dx	83					Other cardiac dx	93					IHD - Acute Myocardial Infarction	No	Ischemic Heart Disease	No
378	M	55.1	Neg	Neg	N19	I10	I12	No	Acute abdomen	53	HIV/AIDS	41			HIV/AIDS	99					Undetermined	No	Undetermined	No
379	F	29.9	Neg	Neg	K25	M05	K25	No	Meningitis/encephalitis	71					HIV/AIDS	93					Undetermined	No	Diarrhea/Dysentery	No
380	M	48.1	Neg	Neg	J18		J18	No	ARTI	100					ARTI	99					Undetermined	No	Undetermined	No
381	F	61.1	Neg	Neg	G40	I64	G40	No	Stroke	100					Stroke	99					Diabetes	No	Stroke	No
382	F	48.1	Neg	Neg	E11		E11	No	Stroke	42	HIV/AIDS	34			Stroke	78					Undetermined	No	Undetermined	No
383	F	48.1	Neg	Neg	I61	I10	I61	No	Stroke	100					Stroke	99					Undetermined	No	Stroke	No
384	F	26.2	Neg	Neg	A41	C83	C85	No	HIV/AIDS	100					HIV/AIDS	99					Leukemia/Lymphomas	No	Breast Cancer	No
385	F	51.9	Neg	Neg	C53		C53	No	Reproductive Ca	98					Reproductive Ca	98					Cervical Cancer	No	Cervical Cancer	No
386	F	60.4	Neg	Neg	C92		C92	No	Digestive Ca	99					Digestive Ca	99					Breast Cancer	No	Leukemia/Lymphomas	No
387	F	43.1	Neg	Neg	I64	I10	I64	No	Asthma	89					Stroke	99					Undetermined	No	Stroke	No
388	M	46.4	Neg	Neg	C14		C97	No	Respiratory Ca	100					Respiratory Ca	99					AIDS	Yes	Esophageal Cancer	No
389	M	48.0	Neg	Neg	N17	A16	N17	No	Pulmonary TB	100					Pulmonary TB	99					Pneumonia	No	TB	No
390	F	57.9	Neg	DK	I61	I10	I61	No	Stroke	100					Stroke	99					Stroke	No	Stroke	No
391	M	61.1	Neg	Neg	N18	E11	E11	No	HIV/AIDS	100					HIV/AIDS	93					Stroke	No	Stroke	No
392	M	27.7	Neg	Neg	C18		C18	No	Digestive Ca	83					Digestive Ca	95					Stomach Cancer	No	Stomach Cancer	No
393	F	60.9	Neg	Neg	C96	I74	C49	No	Digestive Ca	97					Digestive Ca	97					Diarrhea/Dysentery	No	Leukemia/Lymphomas	No
394	M	64.1	Neg	Neg	E11	A09	E11	No	Stroke	50	Digestive Ca	50			Digestive Ca	75					Undetermined	No	Diarrhea/Dysentery	No
395	F	42.4	Neg	DK	K72		K72	No	ARTI	99					ARTI	98					Breast Cancer	No	Breast Cancer	No
396	F	21.4	Neg	Neg	K29		K29	No	Poisoning	94					Poisoning	94					Poisonings	No	Suicide	No

Participant details				VA	PCVA				InterVA-4						InterVA-5						SmartVA-Analyze v1.1.1		SmartVA-Analyze v.1.2.1	
ID	Sex	Age*	HIV status	HIV status	iCoD	uCoD	MMDS CoD	HIV CoD?	Cause 1	Lik 1	Cause 2	Lik 2	Cause 3	Lik 3	Cause 1	Lik 1	Cause 2	Lik 2	Cause 3	Lik 3	CoD	HIV CoD?	CoD	HIV CoD?
397	F	33.8	Neg	Neg	N17	E10	N17	No	Digestive Ca	98					Digestive Ca	90					Undetermined	No	Other Non-communicable Diseases	No
398	M	52.6	Neg	Neg	N18	I10	I12	No	Renal failure	97					Renal failure	97					Epilepsy	No	Undetermined	No
399	M	35.9	Neg	DK	K56		K56	No	Reproductive Ca	94					Reproductive Ca	86					AIDS	Yes	Cirrhosis	No
400	F	22.2	Neg	Neg	E10		E10	No	Other/unspecified infection	67					Other/unspecified infection	86					Epilepsy	No	Diabetes	No
401	M	57.9	Neg	Neg	J18	G40	G40	No	HIV/AIDS	45	Pulmonary TB	29			Indeterminate	0					Undetermined	No	Other Non-communicable Diseases	No
402	M	27.8	Neg	Neg	I74		I82	No	ARTI	100					ARTI	98					Undetermined	No	Undetermined	No
403	F	52.0	Neg	Neg	K72	K83	K83	No	HIV/AIDS	100					Other Ca	87					AIDS	Yes	Undetermined	No
404	F	37.0	Neg	Neg	I11	N18	I13	No	Other cardiac dx	100					Other cardiac dx	99					Undetermined	No	Undetermined	No
405	M	61.8	Neg	Neg	N17		N17	No	ARTI	100					ARTI	95					Undetermined	No	Undetermined	No
406	M	48.6	Neg	Neg	I64		I64	No	Stroke	100					Stroke	99					Stroke	No	Stroke	No
407	M	25.7	Neg	Neg	N19		N19	No	HIV/AIDS	98					HIV/AIDS	99					Undetermined	No	Undetermined	No
408	M	47.4	Neg	DK	D37		D37	No	Digestive Ca	48	Diarrhoeal dx	37			Diarrhoeal dx	64					Undetermined	No	Undetermined	No
409	F	67.0	Neg	Neg	J17	I64	I64	No	Digestive Ca	60	HIV/AIDS	38			Digestive Ca	98					COPD	No	Other Non-communicable Diseases	No
410	M	69.4	Neg	Neg	A19	I50	A19	No	Pulmonary TB	100					Pulmonary TB	99					Undetermined	No	TB	No
411	F	69.9	Neg	Neg	I50	E11	I50	No	Pulmonary TB	76					Other cardiac dx	65					Renal Failure	No	Stroke	No
412	F	51.2	Neg	DK	J90	I64	J90	No	Stroke	100					Stroke	99					Epilepsy	No	Stroke	No
413	F	28.3	Neg	Neg	I50	O99	I50	No	Abortion-related	85					Diabetes	47	Other cardiac dx	46			Maternal	No	Maternal	No
414	M	48.0	Neg	Neg	C61		C61	No	Oral Ca	60					Oral Ca	42	Respiratory Ca	33			Undetermined	No	Undetermined	No
415	M	28.9	Neg	Neg	K52		K52	No	HIV/AIDS	86					HIV/AIDS	55	Severe malnutrition	43			Diarrhea/Dysentery	No	Cirrhosis	No

Participant details				VA	PCVA				InterVA-4						InterVA-5						SmartVA-Analyze v1.1.1		SmartVA-Analyze v.1.2.1	
ID	Sex	Age*	HIV status	HIV status	iCoD	uCoD	MMDS CoD	HIV CoD?	Cause 1	Lik 1	Cause 2	Lik 2	Cause 3	Lik 3	Cause 1	Lik 1	Cause 2	Lik 2	Cause 3	Lik 3	CoD	HIV CoD?	CoD	HIV CoD?
416	F	55.3	Neg	Neg	C53		C53	No	Reproductive Ca	89					Reproductive Ca	89					Undetermined	No	Undetermined	No
417	M	50.9	Neg	Neg	L03	E11	L03	No	Cirrhosis	74					Cirrhosis	86					Undetermined	No	Ischemic Heart Disease	No
418	F	50.0	Neg	Neg	I64		I64	No	Stroke	55	Diabetes	44			Diabetes	96					Stroke	No	Stroke	No
419	M	47.8	Neg	Neg	I50	I25	I25	No	Other cardiac dx	60	Acute cardiac dx	39			Other cardiac dx	91					Stomach Cancer	No	Undetermined	No
420	F	54.3	Neg	Neg	L03	E11	L03	No	Diabetes	67					Stroke	98					Diabetes	No	Stroke	No
421	M	56.5	Neg	Neg	I50		I50	No	Respiratory Ca	75					Other cardiac dx	56	Respiratory Ca	43			Undetermined	No	Undetermined	No
422	M	62.5	Neg	Neg	A16		A16	No	Stroke	70					Digestive Ca	60	Stroke	38			Cirrhosis	No	TB	No
423	M	54.1	Neg	Neg	C80		C80	No	Digestive Ca	99					Digestive Ca	99					IHD - Acute Myocardial Infarction	No	Undetermined	No
424	M	56.8	Neg	Neg	D54		D64	No	Other Ca	47	Pulmonary TB	30			Other Ca	67					Cirrhosis	No	TB	No
425	M	64.0	Neg	DK	J47	I64	J47	No	Diarrhoeal dx	65	Digestive Ca	33			Digestive Ca	95					Diarrhea/Dysentery	No	Stroke	No
426	F	59.0	Neg	Neg	N19		N19	No	Renal failure	89					Renal failure	89					Undetermined	No	Diabetes	No
427	M	24.0	Neg	Neg	G00	B24	B20	Yes	Indeterminate						Indeterminate	0					Undetermined	No	Undetermined	No
428	F	48.3	Neg	Neg	C80		C80	No	Reproductive Ca	100					Reproductive Ca	99					Cervical Cancer	No	Cervical Cancer	No
429	F	69.6	Neg	Neg	C53		C53	No	Digestive Ca	57	Acute abdomen	37			Other cardiac dx	43	Digestive Ca	42			Breast Cancer	No	Breast Cancer	No
430	M	65.1	Neg	Neg	A19		A19	No	Pulmonary TB	96					Pulmonary TB	99					Leukemia/Lymphomas	No	Leukemia/Lymphomas	No
431	F	65.0	Neg	Neg	J18		J18	No	Pulmonary TB	100					Pulmonary TB	99					Pneumonia	No	Other Cardiovascular Diseases	No
432	M	59.5	Neg	Neg	C71		C71	No	Stroke	100					Stroke	99					Stroke	No	Stroke	No
433	F	58.8	Neg	DK	I64		I64	No	Diabetes	92					Diabetes	98					Diabetes	No	Ischemic Heart Disease	No

Participant details				VA	PCVA				InterVA-4						InterVA-5						SmartVA-Analyze v1.1.1		SmartVA-Analyze v.1.2.1	
ID	Sex	Age*	HIV status	HIV status	iCoD	uCoD	MMDS CoD	HIV CoD ?	Cause 1	Lik 1	Cause 2	Lik 2	Cause 3	Lik 3	Cause 1	Lik 1	Cause 2	Lik 2	Cause 3	Lik 3	CoD	HIV CoD ?	CoD	HIV CoD ?
434	M	59.3	Neg	Neg	A16		A16	No	Pulmonary TB	99					Pulmonary TB	93					Undetermined	No	TB	No
435	M	69.1	Neg	Neg	I50		I50	No	COPD	100					COPD	99					COPD	No	Chronic Respiratory	No
436	M	39.0	Neg	Neg	A16		A16	No	Pulmonary TB	100					Pulmonary TB	96					Pneumonia	No	TB	No
437	M	69.3	Neg	Neg	I64		I64	No	Stroke	74					Renal failure	48	Stroke	37			Stroke	No	Stroke	No
438	M	61.6	Neg	DK	A18	G82	A16	No	Digestive Ca	100					Digestive Ca	99					Diabetes	No	TB	No
439	M	59.6	Neg	Neg	I50		I50	No	Pulmonary TB	48	Other Ca	29			Other Ca	59	HIV/AIDS	29			COPD	No	Undetermined	No
440	M	56.3	Neg	Neg	I64	I10	I64	No	Stroke	48	Pulmonary TB	38			Stroke	99					Undetermined	No	Stroke	No
441	M	52.2	Neg	Neg	L22		C24	No	Digestive Ca	100					Digestive Ca	99					Undetermined	No	TB	No
442	F	50.3	Neg	Neg	C76		C80	No	Other Ca	65	Digestive Ca	32			Other Ca	85					Undetermined	No	Undetermined	No
443	M	61.5	Neg	Neg	C61	C79	C61	No	Digestive Ca	88					Other Ca	98					Undetermined	No	Prostate Cancer	No
444	M	57.7	Neg	Neg	N18	E11	E11	No	Other cardiac dx	87					Other cardiac dx	86					Undetermined	No	Ischemic Heart Disease	No
445	M	35.4	Neg	Neg	C34		C34	No	Respiratory Ca	99					Respiratory Ca	99					Undetermined	No	Prostate Cancer	No
446	F	51.0	Neg	Neg	C53		C53	No	Indeterminate	100					Other cardiac dx	43	Other Ca	34			Undetermined	No	Undetermined	No
447	F	45.3	Neg	Neg	D43		D43	No	HIV/AIDS	100					HIV/AIDS	87					Undetermined	No	Undetermined	No
448	M	58.0	Neg	Neg	C34		C34	No	Respiratory Ca	92					Respiratory Ca	99					IHD - Acute Myocardial Infarction	No	TB	No
449	M	56.7	Neg	Neg	K27		K27	No	Digestive Ca	99					Digestive Ca	99					Undetermined	No	Undetermined	No
450	F	52.6	Neg	Neg	A18	I64	A16	No	Pulmonary TB	100					Pulmonary TB	72					IHD - Acute Myocardial Infarction	No	TB	No
451	F	30.3	Neg	Neg	C32		C32	No	Digestive Ca	98					Digestive Ca	88					Stomach Cancer	No	Stomach Cancer	No
452	F	27.3	Neg	Neg	C40		C41	No	Indeterminate	100					Oral Ca	87					Undetermined	No	Leukemia/Lymphomas	No
453	M	62.3	Neg	Neg	K27	E11	K27	No	Pulmonary TB	100					Pulmonary TB	86					Stomach Cancer	No	Chronic Respiratory	No

Participant details				VA	PCVA				InterVA-4						InterVA-5						SmartVA-Analyze v1.1.1		SmartVA-Analyze v.1.2.1	
ID	Sex	Age*	HIV status	HIV status	iCoD	uCoD	MMDS CoD	HIV CoD?	Cause 1	Lik 1	Cause 2	Lik 2	Cause 3	Lik 3	Cause 1	Lik 1	Cause 2	Lik 2	Cause 3	Lik 3	CoD	HIV CoD?	CoD	HIV CoD?
454	F	51.5	Neg	Neg	K56	K36	K56	No	Reproductive Ca	50	Digestive Ca	49			Reproductive Ca	50	Digestive Ca	49			Undetermined	No	Undetermined	No
455	F	61.8	Neg	Neg	C76		C80	No	Digestive Ca	90					Digestive Ca	94					Diarrhea/Dysentery	No	Undetermined	No
456	F	28.4	Neg	Neg	M35		M35	No	Digestive Ca	95					HIV/AIDS	79					Maternal	No	Maternal	No
457	F	24.6	Neg	Neg	N18	O13	I12	No	HIV/AIDS	99					Other cardiac dx	90					Breast Cancer	No	Breast Cancer	No
458	M	61.2	Neg	Neg	C15		C15	No	Digestive Ca	100					Digestive Ca	99					Leukemia/Lymphomas	No	Prostate Cancer	No
459	M	20.2	Neg	Neg	R57	E35	E34	No	Digestive Ca	86					Digestive Ca	93					Colorectal Cancer	No	Other Non-communicable Diseases	No

*Age at death, years

CoD: cause of death; F: female; iCoD: immediate cause of death; IV4: InterVA-4; IV5: InterVA-5; Lik: likelihood; M: male; MMDS: mortality medical data system; neg: negative; PCVA: physician-certified verbal autopsy; pos: Positive; Q: question; SV1.1.1: SmartVA-Analyze v1.1.1; SV1.2.1: SmartVA-Analyze v.1.2.1; uCoD: underlying cause of death; VA: verbal autopsy

Supplementary figure 1. Overview of modified physician-certified verbal autopsy procedure

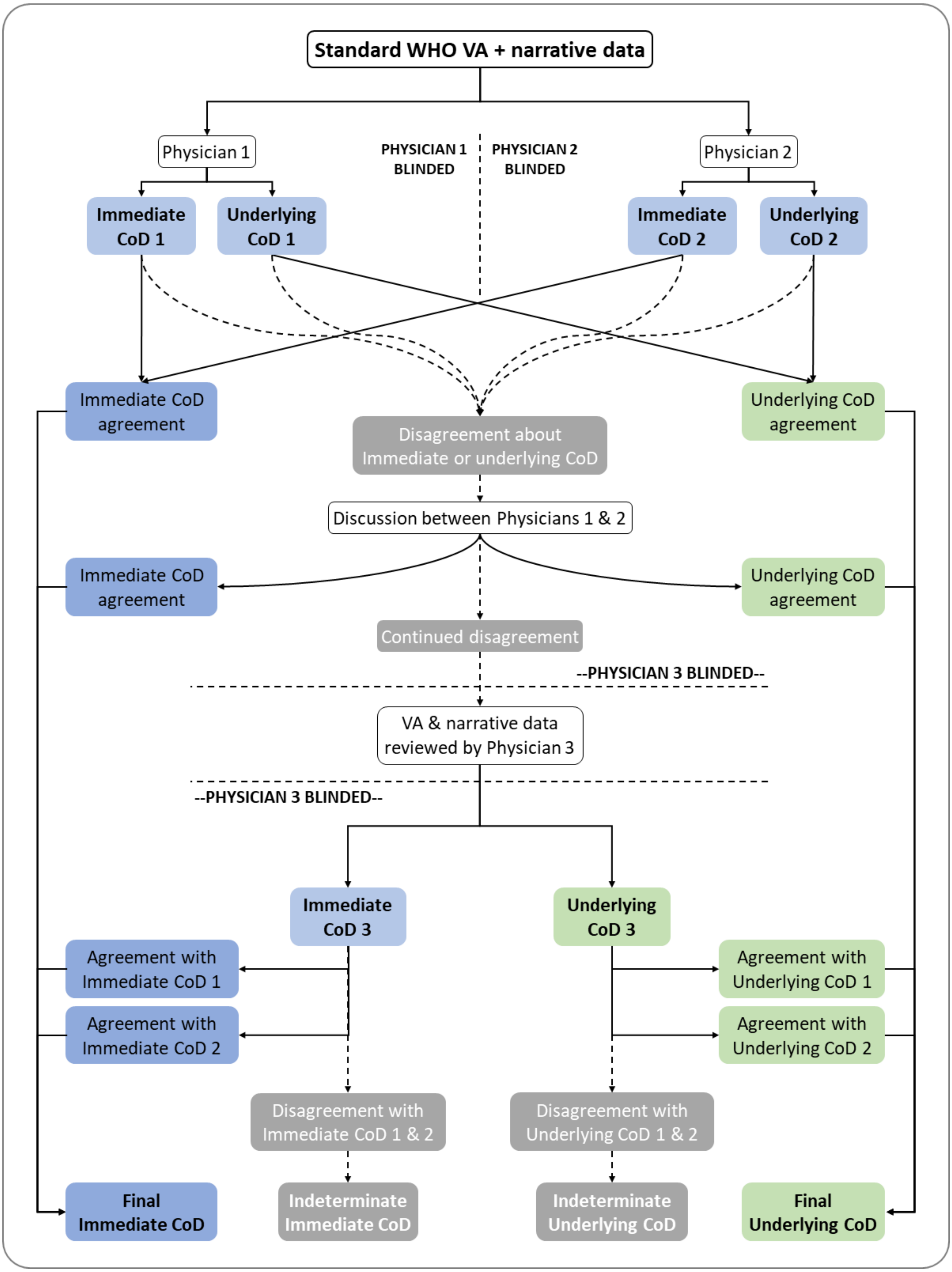

CoD: cause of death; VA: verbal autopsy; WHO: World Health Organization
